# Intervention of oncostatin M-driven mucosal inflammation by berberine exerts therapeutic property in chronic ulcerative colitis

**DOI:** 10.1038/s41419-020-2470-8

**Published:** 2020-04-24

**Authors:** Heng Li, Chunlan Feng, Chen Fan, Yang Yang, Xiaoqian Yang, Huimin Lu, Qiukai Lu, Fenghua Zhu, Caigui Xiang, Zongwang Zhang, Peilan He, Jianping Zuo, Wei Tang

**Affiliations:** 1 Laboratory of Anti-inflammation and Immunopharmacology, Shanghai Institute of Materia Medica, Chinese Academy of Sciences, 201203 Shanghai, China; 2 School of Pharmacy, University of Chinese Academy of Sciences, 100049 Beijing, China; 3 Laboratory of Immunology and Virology, Shanghai University of Traditional Chinese Medicine, 201203 Shanghai, China

**Keywords:** Ulcerative colitis, Chronic inflammation

## Abstract

Ulcerative colitis (UC) is a chronic and etiologically refractory inflammatory gut disorder. Although berberine, an isoquinoline alkaloid, has been revealed to exert protective effects on experimental colitis, the underlying molecular mechanism in chronic intestinal inflammation remains ill-defined. This study was designed to uncover the therapeutic efficacy and immunomodulatory role of berberine in chronic UC. Therapeutic effects of oral administration of berberine were investigated in dextran sodium sulfate (DSS)-induced murine chronic UC and the underlying mechanisms were further identified by si-OSMR transfection in human intestinal stromal cells. Berberine significantly attenuated the experimental symptoms and gut inflammation of chronic UC. Berberine treatment could also maintain the intestinal barrier function and rectify tissue fibrosis. In accordance with infiltrations of antigen-presenting cells (APCs), innate lymphoid cells (ILCs), and activated NK cells in colonic lamina propria, increased expression of OSM and OSMR were observed in the inflamed tissue of chronic UC, which were decreased following berberine treatment. Moreover, berberine inhibited the overactivation of human intestinal stromal cells through OSM-mediated JAK-STAT pathway, which was obviously blocked upon siRNA targeting OSMR. The research provided an infusive mechanism of berberine and illustrated that OSM and OSMR intervention might function as the potential target in chronic UC.

## Introduction

Ulcerative colitis (UC), belonging to one form of inflammatory bowel diseases (IBD), is characterized by severe diarrhea, unintended weight loss, bloody stools, abdominal pain, and fatigue^1–3^. Inflammation in UC is dominantly presented in the mucosal and submucosal layer of colon and rectum^4,5^. The pathogenesis of UC is multidimensional and linked to the coactions between genetic susceptibility, immune system imbalance, dysfunction of intestinal barrier, and turbulence of microorganisms^6,7^. During the progression and exacerbation of UC, colonic tissue fibrosis, as the fact of Sirius red staining and α-SMA^+^ staining, is considered as the key factor, leading to collagen deposition, destruction of extracellular matrix (ECM), and thickening of the intestinal wall^8–10^. The fibrotic development is closely associated with a cascade of processes, including epithelial cells injury and reconstitution, activation of immune cells and mesenchymal cells, as well as angiogenesis and lymphangiogenesis^11^. Moreover, increased deposition of ECM is mainly derived from abnormal activation of fibroblasts, myofibroblasts, and smooth muscle cells in response to gut inflammatory mediators^12^.

Oncostatin M (OSM), a part of the IL-6 cytokine family, is mainly synthesized by activated macrophages, neutrophils, dendritic cells, and T cells^13,14^. Previous studies have verified that recombinant OSM could induce the activation of JAK-STAT (including JAK1, JAK2, STAT1, STAT3, STAT4, STAT5, and STAT6), MAPK, and PI3K-AKT pathways via the heterodimeric receptors comprised of OSMR and gp130^13,15^. OSM is a pleiotropic cytokine, playing a vital part in chronic inflammation, cardiac remodeling, hematopoiesis, liver development, and bone formation^16,17^. Results from the colonic biopsies from IBD patients demonstrated that OSM and OSMR were highly expressed and positively correlated with the disease severity of Crohn’s diseases (CD) and UC^15^. In the intestinal mucosa, OSMR was mainly expressed on stromal cells, not immune cells and epithelial cells, and redundant OSM further led to numerous inflammatory cells infiltrating to the mucosa, which pathologically contributed to intestinal fibrosis^13,15^. Targeting OSM and OSMR may provide new sights for understanding the underlying pathomechanism of UC and serve as an alternative therapeutic strategy^18,19^.

Berberine, an alkaloid bearing the characteristic skeleton of quaternary ammonium, is primarily derived from the roots, stems, rhizomes, or bark of certain plants, such as *Coptis chinensis* (Chinese goldthread), *Berberis vulgaris* (barberry), *Argemone mexicana* (prickly poppy), and *Hydrastis Canadensis* (goldenseal)^20^. These herbs have been widely used in China for centuries in the treatment of diarrhea, abdominal pain, and gastroenteritis. As a dazzling compound with the potential in treating many diseases, berberine is undoubtably among the most thoroughly studied natural products in the world. Increasing evidence has suggested that berberine possessed numerous pharmacological activities, including anti-microbial, anti-diabetic, anti-colorectal cancer, analgesic, lipid modulatory, anti-depressant, and blood glucose and anti-inflammatory effects^21^. Particularly, berberine has been suggested to function as an effective agent in treating experimental colitis, including UC and CD, which were largely due to the role of berberine in modulating gut microbiota, neurogenic inflammation, mucosal immunity, and barrier function^20,22–24^. Though the underlying mechanism of berberine has been focused on activation of adenosine 5′-monophosphate (AMP)-activated protein kinase (AMPK)^23^, we revealed a new molecular mechanism of berberine in attenuating chronic UC.

In the present study, we aimed to investigate the immunoregulatory role and underlying molecular mechanism of berberine in dextran sodium sulfate (DSS)-induced murine chronic colitis. Herein, we demonstrated that berberine effectively ameliorated disease severity and rectified gut intestinal barrier disturbance and colonic fibrosis through suppressing OSM-driven mucosal inflammation. Our study provided a novel and infusive mechanism of berberine and suggested OSM and OSMR intervention might function as the potential target in chronic mucosal inflammation.

## Materials and methods

### Animals

All applicable institutional and/or national guidelines for the care and use of animals were followed. Wild-type male C57BL/6 mice (8 weeks, 22–24 g) obtained from Shanghai Laboratory Animal Center of the Chinese Academy of Sciences were used for this investigation. All experiments were carried out according to the National Institutes of Health Guide for Care and Use of Laboratory Animals and were approved by the Bioethics Committee of the Shanghai Institute of Materia Medica (SIMM). Mice were housed under specific pathogen-free conditions with 12 h of light/12 h of dark cycle, 22 ± 1 °C and 55 ± 5% relative humidity. All mice were fed standard laboratory chow and water ad libitum and allowed to acclimatize in our facility for 1 week before any experiments started.

### Induction and assessment of DSS-induced chronic colitis

To evaluate the damage progression overtime, experimental chronic colitis was induced by giving mice three cycles of DSS (MP Biomedicals, CA, USA) according to the previous description^25,26^. Briefly, each cycle consisting 2% DSS for 7 days followed by sterile drinking water for 14 days. The first and last day of experiment were designated as day 1 and day 50, respectively. All mice were randomly divided into three groups consisting of normal, vehicle (only DSS) and berberine treatment (DSS plus berberine, Sigma-Aldrich, St. Louis, MO, USA) with 15 mice per group. Berberine (50 mg/kg) was dissolved in sterile water and orally administrated daily from day 15 on. Body weight change, stool consistency and occult blood, as indicators of disease activity index (DAI), were blind monitored by three investigators according to the described criterion^26^. Briefly, body weight loss of 1–5%, 5–10%, 10–20%, and >20% was scored as 1, 2, 3, and 4, respectively. For stool consistency, 0 was scored for normal-formed pellets, 1 for soft but formed stools, 2 for soft stools, 3 for very soft and wet stools, and 4 for watery diarrhea. Bleeding stools were scored 0 for no blood in hemoccult, 1 for weakly positive hemoccult, 2 for positive hemoccult, 3 for blood traces in stool visible, and 4 for gross bleeding from the rectum. The DAI was calculated as the total of these scores ranging from 0 (no inflammation) to 12 (severe colitis).

By the end of treatment, animals were sacrificed and serum samples were collected for biochemical indexes measurement using a HITACHI-7080 automatic biochemical analyzer (Hitachi High Technologies Corporation, Tokyo, Japan). The colons were removed followed by weight and length records and 1-cm segments from the same regions of the colons were washed with PBS (phosphate-buffered saline), cut into three defined biopsies and then cultured for 24 h containing 0.5 ml of RMPI-1640 media (Gibco, Grand Island, NY, USA) containing 10% FBS (Hyclone, South Logan, UT, USA) at 37 °C in a humidified incubator of 5% CO_2_. The supernatants were collected and determined for cytokines production.

### Histological analysis

Colon sections were fixed in 10% phosphate-buffered saline (PBS)-buffered formalin, embedded in paraffin and then stained with hematoxylin and eosin (H&E). Histopathological examination was observed under a light microscope and scored blind by three investigators in the Center for Drug Safety Evaluation and Research, SIMM, Chinese Academy of Sciences (CAS), which are authoritative under Good Laboratory Practice. A scoring system was presented as: 0, no evidence of inflammation; 1, low level inflammation with scattered mononuclear cells (1–2 foci); 2, moderate inflammation with multiple foci of mononuclear cells; 3, high level inflammation with increased vascular density and marked wall thickening; and 4, maximal inflammation with transmural leukocyte infiltration and loss of goblet cells. For colon fibrosis, paraffin-embedded colonic sections were subjected to dehydration and incubated with picrosirius red solution. The positive staining for collagen depositions were acquired under a microscope (Olympus IX73, Tokyo, Japan).

### In vivo living imaging and intestinal permeability measurement

The luminol-based chemiluminescent probe L-012 sodium (Novus Biologicals, Littleton, CO, USA) and the fluorescent chemical FITC-dextran (Sigma-Aldrich) were used for in vivo imaging to reveal the intestinal inflammation and intestinal retention, respectively^27,28^. IVIS Spectrum CT system was initiated according to the manufacturer’s instructions. Briefly, animals in each group were anesthetized with 1.5–2.0% isoflurane, injected intraperitoneally with L-012 (25 mg/kg) and the bioluminescent images were obtained using the autoexposure option to automatically optimize signal intensity. Alternatively, mice were fasted overnight and FITC-dextran (600 mg/kg) were orally administrated and then after 4 h distribution, they were anesthetized and exposed to the IVIS Spectrum CT system. The fluorescent images were obtained at 480 nm excitation and 520 nm emission. Meanwhile, serum samples of all mice were collected and serum fluorescence intensity were measured at 480 nm excitation and 520 nm emission using a microplate reader (Spectramax M5, Molecular Devices Corporation, Sunnyvale, CA).

### Transmission electron microscopy (TEM) and morphometry

At the end of treatment, mice were euthanized and colonic sections were carefully dissected and fixed in 2.5% glutaraldehyde in PBS (Sigma-Aldrich) at 4 °C. Sections were then post-fixed with 1% OsO_4_ (Sigma-Aldrich) for 1 h and embedded in resin. Ultra-thin sections (70 nm) in each group were prepared and stained as previous description^29^. The structures of colonic sections were observed using TEM (Philips Tecnai 20 U-Twin, Holland).

### Ex vivo induction and CD4^+^ T cells purification from spleens and MLNs

Splenocytes and mesenteric lymph nodes (MLNs) cells were prepared and activated with anti-CD3 antibodies (5 μg/ml) or LPS (10 μg/ml), respectively. Cells were added with 0.5 μCi/well [^3^H-TdR] thymidine to determine proliferation activity and the supernatants were used to detect cytokines.

Polyclonal CD4^+^ T cells were purified from splenocytes and MLNs cells using EasySep™ mouse CD4^+^ T Cell isolation kit (Stemcell, Vancouver, BC, Canada) according to the instructions. The purity of the CD4^+^ T cells was consistently >98%, assayed by flow cytometry. CD4^+^ T cells were incubated with berberine at the indicated concentrations for 48 h and then 20 μl of CCK-8 reagent (Dojindo, Kumamoto, Japan) was added to determine the cell viability. Moreover, cells were stimulated with anti-CD3 antibodies (5 μg/ml) and anti-CD28 antibodies (2 μg/ml) for 48 h. Subsequently, the cells were pulsed with 0.5 μCi/well [^3^H-TdR] thymidine to determine cells proliferation activity and the cell supernatants were collected to quantify cytokines production.

### Isolation of lamina propria cells and flow cytometry analysis

Single-cell suspensions from the colonic lamina propria were prepared as previously described^30,31^. Colon samples in each group were cut into small pieces and incubated in RPMI-1640 containing 10% FBS and 5 mM EDTA for 15 min and then digested using 0.5 mg/ml Type IV collagenase, 3 mg/ml dispase II, and 0.1 mg/ml DNase I in a 37 °C shaking incubator for 30 min. Single cells were filtered using a 70 μm filter and then washed twice with PBS. Cells were further incubated with FVD eFluor™ 780 (eBioscience, San Diego, CA, USA) to identify viable cells and blocked by conjugating with 2.4G2 (eBioscience). The cell surfaces were stained with brilliant ultraviolet 395 (BUV395), fluorescein isothiocyanate (FITC), phycoerythrin (PE), peridinin-chlorophyll proteins-Cyanine5.5 (Percp-Cy5.5), allophycocyanin (APC), brilliant violet 421 (BV421)-labeled antibodies. For intracellular staining, cells were fixed and permeabilized using Transcription Factor Buffer Set (BD Biosciences, San Jose, CA, USA). Then, cells were labeled intracellularly with FITC-conjugated anti-T-bet, APC-conjugated anti-GATA3, and BV421-conjugated RORγT. All antibodies in this research were purchased from BD Biosciences or Thermo Fisher Scientific (Waltham, MA, USA). Data were analyzed using FlowJo software (Tree Star, Ashland, OR, USA).

### Cell cultures and in vitro stimulation

Human intestinal stromal cell line, CCD-18Co cells, human myeloid leukemia cell line, U937 cells, human monocytic cell line, THP-1 cells, and human immortalized T lymphocyte cell line, Jurkat T cells, were obtained from American Type Culture Collection (ATCC, Manassas, VA, USA). All cell lines were authenticated by STR profiling and tested for mycoplasma contamination. CCD-18Co cells were maintained in DMEM containing 10% FBS, 2 mmol/l of l-glutamine, 100 U/ml penicillin, and 100 μg/ml streptomycin. CCD-18Co cells were incubated with berberine at the indicated concentrations for 24 h and then 20 μl of CCK-8 reagent (Dojindo) was added to determine the cell viability. U937 cells, THP-1 cells, and Jurkat T cells were cultured in RMPI-1640 media containing 10% FBS, 2 mmol/l of l-glutamine, 100 U/ml penicillin, and 100 μg/ml streptomycin. The cells were maintained at 37 °C in a humidified incubator of 5% CO_2_. Human CCD-18Co cells were adhered overnight and the media were removed. Cells were preincubated with berberine at the indicated concentrations for 1 h, followed by recombinant human OSM (10 ng/ml) for additional 1 or 24 h for the following western blot assay and 2 h for RT-PCR assay.

Bone marrow-derived macrophages (BMDMs) were differentiated from the femur and tibia bones of C57BL/6 mice as previous report^32^. Briefly, BMDMs were separated and cultured for 7 days in Iscove’s Modified Dulbecco’s Medium (IMDM) containing 10% FBS containing 10 ng/ml of mouse colony-stimulating factor (M-CSF, PeproTech, Rocky Hill, NJ, USA). The purity of macrophages (CD11b^+^F4/80^+^) was consistently >98% by flow cytometry analysis. Bone marrow-derived dendritic cells (BMDCs) were prepared as previously described^33^. BMDCs, from C57BL/6 mice, were differentiated in RMPI-1640 media containing 10% FBS containing 15 ng/ml of GM-CSF (PeproTech) and 10 ng/ml of IL-4 (PeproTech). The purity of BMDCs (CD11b^+^CD11c^+^) was consistently >95%, determined by flow cytometry. Differentiated BMDMs and BMDCs were incubated with berberine at the indicated concentrations for 24 h and then 20 μl of CCK-8 reagent (Dojindo) was added to determine the cell viability. Moreover, cells were treated with the indicated concentrations of berberine in the presence of LPS (1 μg/ml) for 24 h.

### SiRNA transfection in human stromal cells

Human CCD-18Co cells were adhered overnight and the media were removed. To knockdown OMSR expression in CCD-18Co cells, three siRNA sequences targeting for OSMR silence were designed and used following the manufacturer′ instructions. Cells were transfected with si-OSMR, mixed in Lipofectamine® RNAiMAX Reagent (Thermo Fisher Scientific). Cells were collected and assayed for OSMR expression after 72 h of transfection using RT-PCR and western blot.

### Cytokines detection by Luminex assay and ELISA

Cytokines level in colonic tissue homogenates and culture supernatant were measured by mouse TNF-α, IFN-γ, IL-2, IL-4, IL-6, IL-10, IL-12p40, and IL-17A ELISA kits (BD Pharmingen, San Diego, CA, USA), mouse IL-1β, IL-5, IL-22, IL-23 ELISA kits (Thermo Fisher Scientific), mouse OSM, IL-13 ELISA kits (R&D, Minneapolis, MN, USA), and human CXCL10 kits (BioLegend, San Diego, CA, USA) according to the manufacturer’s instructions. Cytokines in serum were quantified by Luminex x-MAP technology (Luminex Corp, Austen TX, USA) on a Luminex 200 instrument (Merck Millipore, Billerica, MA, USA). All standard curves with four-parameter logistic fitting, supplied by the manufacturer, had *R*^2^ values at or close to 1. Cytokines concentration were obtained from the standard curves.

### In vitro leukocytes adhesion and chemotaxis

Human CCD-18Co cells were incubated with berberine, 1 h before addition of OSM (10 ng/ml) for 24 h. Human U937, THP-1, and Jurkat T cells were stained with 1 μM calcein AM (BD Biosciences) for 30 min at room temperature and then added onto CCD-18Co monolayer cells and adhered for 30 min at 37 °C in a humidified incubator of 5% CO_2_. Non-adherent cells were washed away with PBS and the calcein AM-labeled cells with green fluorescence were monitored using a fluorescent microscope (Olympus IX73).

For in vitro chemotaxis, the above supernatants from OSM-stimulated CCD-18Co cells were plated into the lower chamber of trans-well plates (Corning Incorporated, Corning, NY, USA), meanwhile, Calcein AM-labeled U937, THP-1, and Jurkat T cells were added into the upper chamber for 2 h incubation. Subsequently, cells in the lower chamber were amounted using hemocytometer (Thermo Fisher Scientific) and photographed under the fluorescent microscope (Olympus IX73).

### Immunohistochemistry and immunofluorescence measurement

Paraffin-embedded colonic slices were dewaxed and rehydrated, followed by blocking with 3% H_2_O_2_ to avoid endogenous peroxidase activity. The masked antigens in cell surface and intracellular regions were retrieved by citrate buffer solution. For immunohistochemistry, colonic sections were incubated overnight with α-SMA (Cell Signaling Technology, Danvers, MA, USA) and then detected by biotinylated horse anti-rabbit secondary antibody, visualized with streptavidin-horseradish peroxidase. The representative images were obtained under the fluorescent microscope (Olympus IX73). For immunofluorescent determination, the colonic tissues were stained with Alexa Fluor 488-conjugated anti-E-cadherin (Cell Signaling Technology), Occludin (Abcam, Cambridge, MA, USA), ZO-1 (Proteintech, Rosemont, USA), FITC-conjugated CD11b (Abcam), ICAM-1 (Abcam), Alexa Fluor 647-conjugated Ly6G (Biolegend), FITC-conjugated CCR5 (Abcam), OSMR (Thermo Fisher Scientific), and p-STAT3 (Cell Signaling Technology). For CCD-18Co cell samples, cells grown on the glass slides, were fixed, blocked with 1% BSA containing 0.1% triton, and stained with p-STAT1 (Cell Signaling Technology) overnight at 4 °C. Unconjugated fluoresceins were further conjugated with FITC anti-rabbit or rat secondary antibodies and then counterstained with DAPI. The representative images were obtained under a Leica TCS SPS microscope (Wetzlar, Germany).

### RNA extraction, cDNA synthesis, and qPCR

Colonic tissues were disrupted using lysis beads in TRIzol extraction reagents (Tiangen, Beijing, China) and Total RNA from colonic homogenates and CCD-18Co cells were isolated using RNAsimple total RNA kit (Tiangen) and followed by reverse transcription using Hifair^TM^ 1st Strand cDNA Synthesis SuperMix (Yeasen, Shanghai, China). Real-time PCR was performed with SYBR® Green Real-time PCR Master Mix (Yeasen) by the Applied Biosystems 7500 Fast Real-Time PCR System (Foster city, CA, USA). The primers for PCR amplification were listed in Supplementary Table S1. ALL expression levels were normalized to an internal housekeeping gene (GAPDH for human samples and β-actin for mouse samples) using the ΔΔ Ct method.

### Total protein extraction and western blot assays

Total protein extracts were prepared with sodium dodecyl sulfate buffer and uniformed by the Pierce BCA protein assay kit (Thermo Fisher Scientific). Equal protein amounts were subjected to 10% SDS–PAGE and transferred to a nitrocellulose membrane (Bio-Rad, Hercules, CA, USA). The membranes were blocked with SuperBlock™ T20 (PBS) blocking buffer (Thermo Fisher Scientific) and then incubated overnight at 4 °C with rabbit, mouse or rat primary antibodies (Supplementary Table S2). The signals were analyzed with HRP-conjugated anti-rabbit IgG (Bio-Rad), HRP-conjugated anti-mouse IgG (Kangchen, Shanghai, Chinia), or HRP-conjugated anti-rat IgG (Cell Signaling Technology) and further visualized by SuperSignal^TM^ West Femto Maximum Sensitivity Substrate (Thermo Fisher Scientific) under ChemiDoc™ MP Imaging System (Bio-Rad).

### Statistical analysis

All data were presented as mean ± SEM. Data from animal studies were subjected to statistical analysis with the sample size of *n* = 15; data from in vitro studies with the sample size of *n* = 3. Statistical differences were detected by GraphPad Prism 6.0 (La Jolla, CA, USA) using a two-tailed Student’s *t*-test or one-way ANOVA with Dunnet’s multiple comparisons test with no significant variance inhomogeneity (*F* achieved *p* < 0.05). *p*-values of <0.05 (p < 0.05) were considered statistically significant.

## Results

### Berberine alleviated the inflammatory injury of DSS-induced chronic colitis

**Fig. 1:**
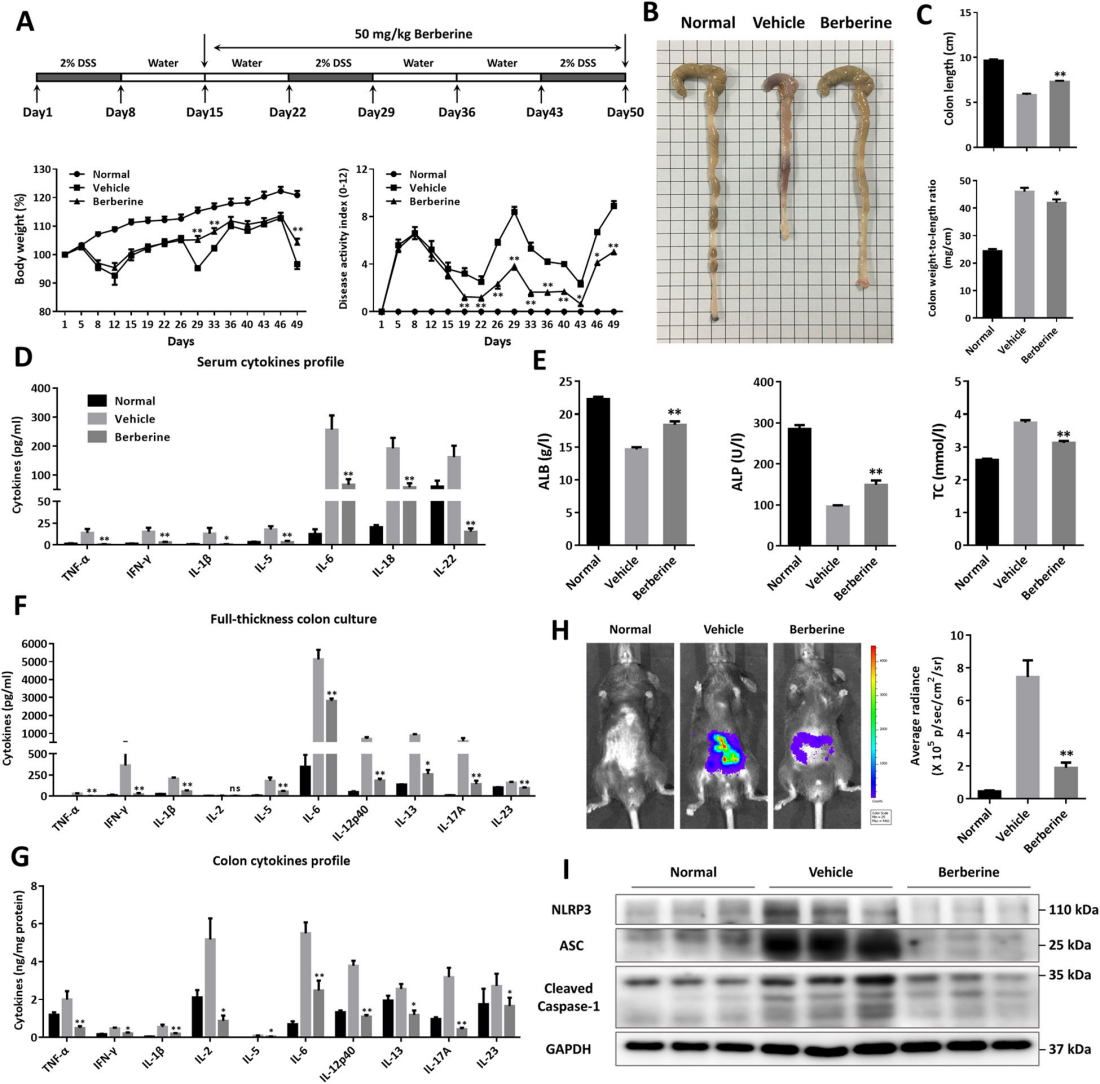
Berberine ameliorated the experimental symptoms and gut inflammation in DSS-induced murine chronic UC. **a** Berberine (50 mg/kg) were orally administrated to DSS-induced UC mice (from day 15 to the end of experiment) and normal control mice were given normal drinking water only. Body weight change (% initial body weight) and disease activity index (DAI) were monitored at the indicated time points. **b** Representative images of colon morphology. **c** Quantitative colon length and the ratio of the colon weight to the length. **d** Serum cytokines determined by Luminex assay. **e** Serum biochemical indicators, including ALB, ALP, and TC. **f** Cytokine secretion levels in full-thickness colon tissue culture. **g** The protein levels of cytokines in colonic homogenates. **h** Representative images of bioluminescent imaging with L-012 and quantitative analysis of positive signals using an IVIS Spectrum CT system. **i** The expression of inflammasome, including NLRP3, ASC, cleaved caspase-1, assayed by western blot and GAPDH was used as a loading control. Data were presented as the mean ± SEM, and *n* = 15 mice per group. *p < 0.05 and **p < 0.01, compared with the vehicle group, were measured by one-way ANOVA.

In order to investigate the therapeutic capacity of berberine in chronic UC, experimental colitis was established by giving mice three cycles of 2% DSS in drinking water and berberine hydrochloride at the dose of 50 mg/kg was orally administrated daily from day 15 to the end point (Fig. 1a). As illustrated in Fig. 1a, marked body weight loss and increased disease activity index (DAI) developed in vehicle-treated mice. In contrast, berberine potently ameliorated the experimental symptoms of chronic UC, manifested with the facts of reversed body weight loss, diarrhea, and stool bleeding (Fig. 1a). Moreover, the colon lengths of berberine-treated mice were significantly longer than those of vehicle-treated mice (Fig. 1b, c). Accompanied by DSS repeated exposure and severe bleeding, multiple inflammatory cytokines in serum, measured by Luminex assay, were upregulated in vehicle mice and decreased markedly upon berberine treatment (Fig. 1d). Owing to excessive hematochezia and diarrhea, loss of serum proteins and metabolic disturbance occurred in UC^34,35^. We further determined some indices of blood biochemistry, including ALB, ALP, and TC, and to some extent, berberine could rectify these biochemical indices, referring to increasing the level of ALB and ALP and reducing the level of TC (Fig. 1e).

To gain evidence whether berberine attenuated the inflammatory conditions within gut microenvironment, we measured a panel of cytokines from the inflamed colonic tissue. As shown in Fig. 1f, the secretory capacity from full-thickness colon culture was much lower in berberine-treated mice, compared to that in vehicle mice (Fig. 1f). Correspondingly, the cytokines profile in colonic homogenates indicated that berberine reduced the protein levels of certain cytokines (Fig. 1g). In addition, in vivo living imaging with L-012 probe further suggested serious inflammation signals in the localization of intestinal tract in vehicle mice, which appeared much weaker following berberine treatment (Fig. 1h). Inflammasome activation functions as an indispensable factor in the pathological process of IBD, contributing to intestinal mucosal inflammation^36^. Interestingly, berberine could suppress the expression level of NLRP3, ASC, and cleaved caspase-1 (Fig. 1i), consistent with the effects of berberine on IL-1β and IL-18 expression (Fig. 1g). Taken together, our results explicated that berberine significantly inhibited the development and severity of chronic intestinal inflammation.

### Berberine maintained the intestinal barrier function accompanying with upregulating the expression of tight junctions

**Fig. 2:**
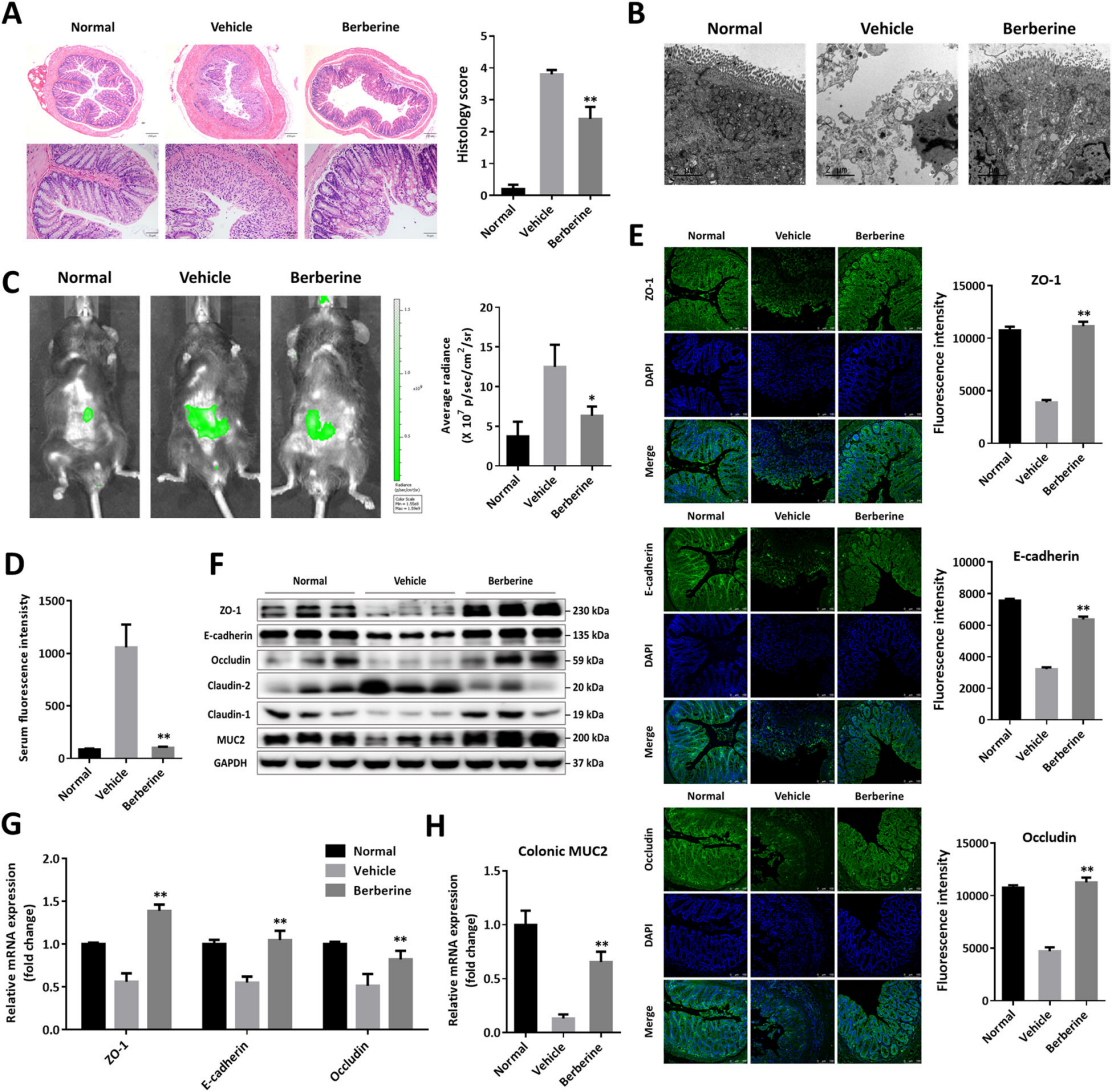
Berberine maintained intestinal mucosal barrier function through upregulating the expression of tight junctions. **a** Representative images of H&E staining of colon sections (×50 and ×100 magnification) and histological scores. **b** Representative images of ultrastructural examination of intestinal epithelial layer by transmission electron microscopy (TEM). **c** Representative images of in vivo imaging with FITC-dextran administration and quantitative assay of imaging with FITC-dextran. **d** Serum fluorescent intensity of FITC-dextran. **e** Colonic sections were immunofluorescent staining with ZO-1, E-cadherin, and occluding, and the nuclei were visualized by DAPI staining. **f** Western blot assay of tight junction proteins, claudins, and MUC2 expression. **g** Gene expression level of tight junction proteins by RT-PCR. **h** Gene expression level of MUC2 by RT-PCR. Data were presented as the mean ± SEM, and *n* = 15 mice per group. *p < 0.05 and **p < 0.01, compared with the vehicle group, were measured by one-way ANOVA.

The intestinal wall, comprising specialized colonocytes with diverse functions, serves as the physical and chemical barrier against exogenous antigens and damage from gut pernicious bacteria^37^. Histologically, DSS induced severe epithelial damage, goblet cells and crypts loss, dense inflammatory cells infiltration, and mucosal ulcers, whereas, berberine largely prevented colonic tissue injury (Fig. 2a). Further TEM analysis of colonic sections revealed significant morphological changes were observed in the intestinal epithelium of vehicle mice. In particular, the affected epithelial cells in vehicle mice are characterized by a reduction or loss of microvillar structures, disintegration of cytoskeleton, swollen mitochondria, increased organelle vacuoles, and enlarged tight junctions (Tj) when compared with those of normal mice (Fig. 2b). The damage resulting from DSS exposure was obviously restored following berberine treatment (Fig. 2b). Moreover, intestinal permeability was also performed by oral administration of FITC-dextran^28^. As presented in Fig. 2c, intestinal injury was consistent with the retention of green fluorescent signals in the abdominal regions. Berberine could reduce the aggregation of FITC-dextran (Fig. 2c) and the serum fluorescent intensity after absorption, distribution, and permeation for 4 h (Fig. 2d). Correspondingly, by contrast to normal mice, significant reductions of colonic Tj proteins, ZO-1, E-cadherin, and occludin, were observed in vehicle mice by immunofluorescent staining (Fig. 2e) and berberine showed quality to increase both the protein (Fig. 2f) and mRNA level (Fig. 2g) of ZO-1, E-cadherin, and occludin. Meanwhile, berberine could also modulate the expression of claudins, as evidence by increased claudin-1 and decreased claudin-2 (Fig. 2f). Furthermore, mucin 2, also known as MUC2, is particularly prominent in the intestine and secreted from goblet cells, contributing to host–microbial interactions and the chemical barrier in gut^38^. In keeping with loss of goblet cells (Fig. 2a), reduced MUC2 in colonic homogenates was monitored in vehicle mice and obviously reversed upon berberine treatment (Fig. 2f, h).

### Berberine attenuated intestinal fibrosis through interfering with OSM-mediated signaling in DSS-induced chronic colitis

**Fig. 3:**
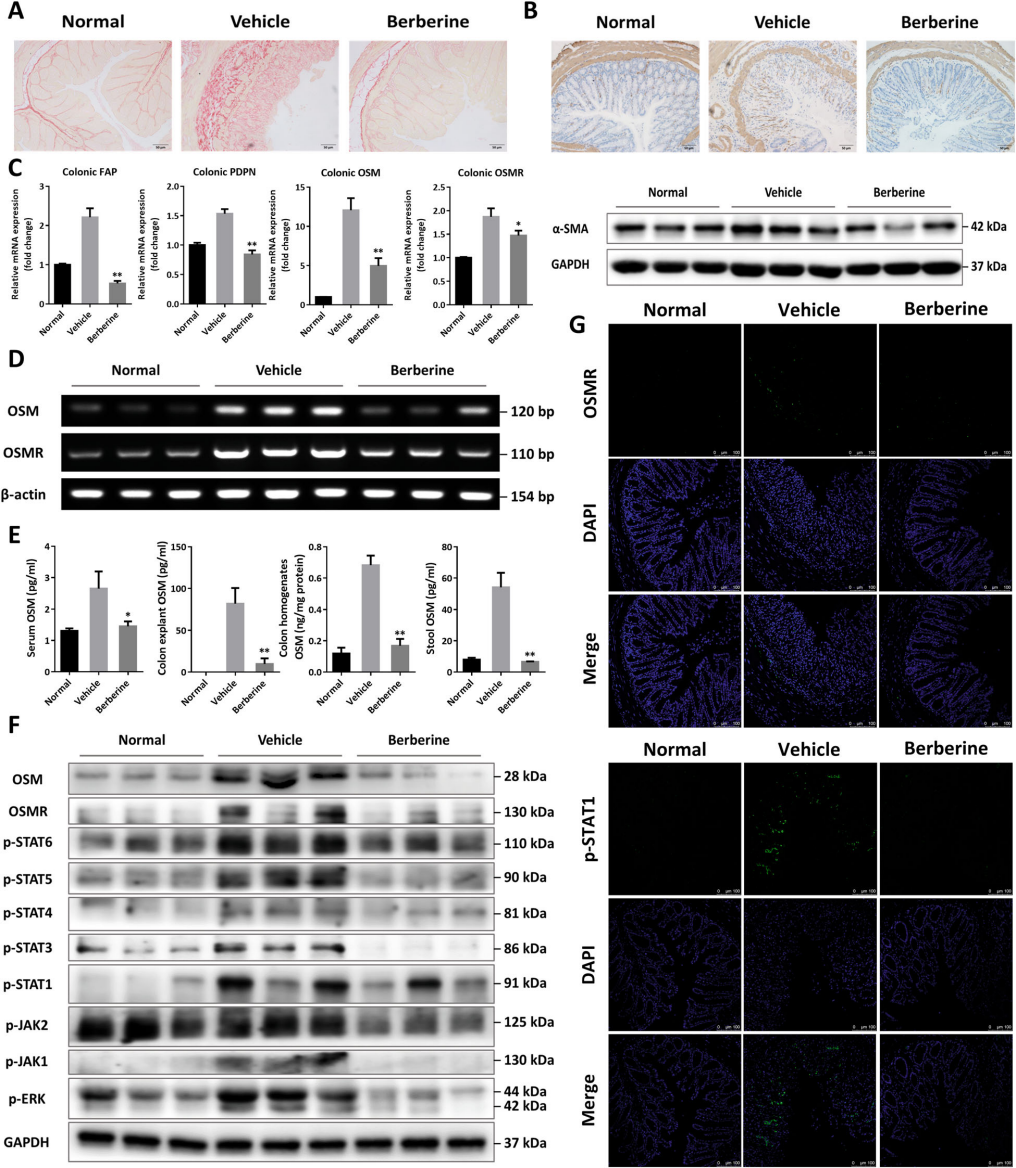
Berberine suppressed intestinal tissue fibrosis through interfering with OSM-mediated signaling pathway. **a** Representative images of picrosirius red staining of colonic tissue. **b** Representative images of immunohistochemistry staining with α-SMA (*upper*) and western blot assay of α-SMA expression (*bottom*). **c** Gene expression of OSM, OSMR, FAP, and PDPN by RT-PCR. **d** The expression level of OSM and OSMR, visualized and separated by agarose gel. **e** OSM levels in serum, full-thickness colon culture, colonic homogenates, and fecal extracts by ELISA. **f** Western blot assay of the expression level of OSM, OSMR, and OSM-mediated downstream signaling pathway. **g** Representative images of immunofluorescence staining with OSMR and p-STAT1. Data were presented as the mean ± SEM and *n* = 15 mice per group. *p < 0.05 and **p < 0.01, compared with the vehicle group, were measured by one-way ANOVA.

In the progression and deterioration of UC, acute colitis caused by DSS definitely progressed to severe chronic colon inflammation with irregular epithelial structure, thickening of gut wall, numerous infiltrates of mononuclear cells, and persistent deposits of collagen^9,39^. Fibrosis-associated mucosal and submucosal collagen depositions in vehicle mice were visualized by picrosirius red staining of colonic tissue and berberine exerted protective effects against collagen depositions (Fig. 3a), which were alternatively confirmed by immunohistochemistry staining and western blotting assay of α-smooth muscle actin (α-SMA, Fig. 3b). Conformably, berberine could also downregulate the expression of fibroblast activation protein-α (FAP), as well as the lymphoid-tissue-like stromal markers podoplanin (PDPN) (Fig. 3c). In the previous reports, OSM and its receptor OSMR expression were positively correlated to FAP and PDPN in the colonic biopsies^15^. Results in Fig. 3c disclosed that both OSM and OSMR were highly expressed in the colonic tissue of vehicle mice, which were in line with the results in the biopsies of patients suffering from IBD. Interestingly, berberine reduced the expression level of OSM and OSMR (Fig. 3c–g). Moreover, we observed that OSMR were highly expressed in the position of colonic stromal cells (Fig. 3g). To further gain evidence toward the effects of berberine on OSM production, we detected the secretion level of OSM in multiple sources. As illustrated in Fig. 3e, OSM protein was readily detectable and highly increased in serum, colonic explant supernatants, colonic tissue homogenates, and fecal matter of vehicle mice. Considering OSM is mainly synthesized by T cells and antigen-presenting cells (APCs), we next measured OSM level in the ex vivo culture of splenocytes, MLNs, purified CD4^+^ T cells from splenocytes and MLNs, as well as in vitro culture of bone marrow-derived macrophages (BMDMs), bone marrow-derived dendritic cells (BMDCs), and purified CD4^+^ T cells from naive C57BL/6 mice (Supplementary Fig. S1). Collectively, berberine dramatically reduced the production of OSM from circulatory system, inflamed tissue, and overactivated immune organs, compared with the vehicle group (Fig. 3e, and Supplementary Fig. S1). OSM augment elicited the phosphorylation of JAT-STAT and MAPK signaling, which contributed to intestinal inflammation^15^. In the present research, DSS led to phosphorylation of JAK1, JAK2, STAT1, STAT3, STAT4, STAT5, STAT6, and ERK (Fig. 3f) with none impacts on the expression the total protein (data not shown). Immunofluorescent staining of p-STAT1 further confirmed the findings (Fig. 3g). Moreover, flow cytometry assay of the colonic lamina propria also demonstrated that berberine could suppress the phosphorylation of STAT1 and STAT3 on the CD3^+^ and CD11b^+^ cells (Supplementary Fig. S2).

### Berberine interfered with the interaction between human intestinal stromal cells and immune cells, in which OSMR functioned as an indispensable element

In the previous research, flow cytometry analysis has revealed that OSMR was mainly expressed by the majority of stromal cells and expressed in low amounts by endothelial cells in the intestinal mucosa^15^. To investigate the underlying molecular mechanism of berberine on OSM/OSMR interactions, we next used primary human intestinal stromal cell, CCD-18Co, to evaluate the recruitment of immune cells following OSM-driven stromal cell activation. As demonstrated in Supplementary Fig. S3a, b, berberine could dose-dependently suppressed human immune cells, U937, THP-1, and Jurkat T cells, adhering onto the monoculture of CCD-18Co cells in the presence of OSM with no cytotoxicity. Besides, upon berberine incubation, there were reduced amounts of migrated immune cells to the lower chambers containing the supernatants from OSM-stimulated CCD-18Co cells (Supplementary Fig. S3c, d). Correspondingly, OSM addition could increase the expression of ICAM-1, while, berberine significantly inhibited the expression of ICAM-1 (Supplementary Fig. S3e). Similarly, berberine downregulated the expression of certain chemokines, including CCL2, CCL3, CCL4, CCL17, CCL20, CXCX9, CXCL10, and CXCL11, in a dose-dependent manner (Supplementary Fig. S3e). In line with the results in Fig. 3e, OSM obviously elicited the phosphorylation of STAT1, STAT3, AKT, and ERK (Supplementary Fig. S3f), whereas, TNF-α triggered little changes in CCD-18Co cells (data not shown).

**Fig. 4:**
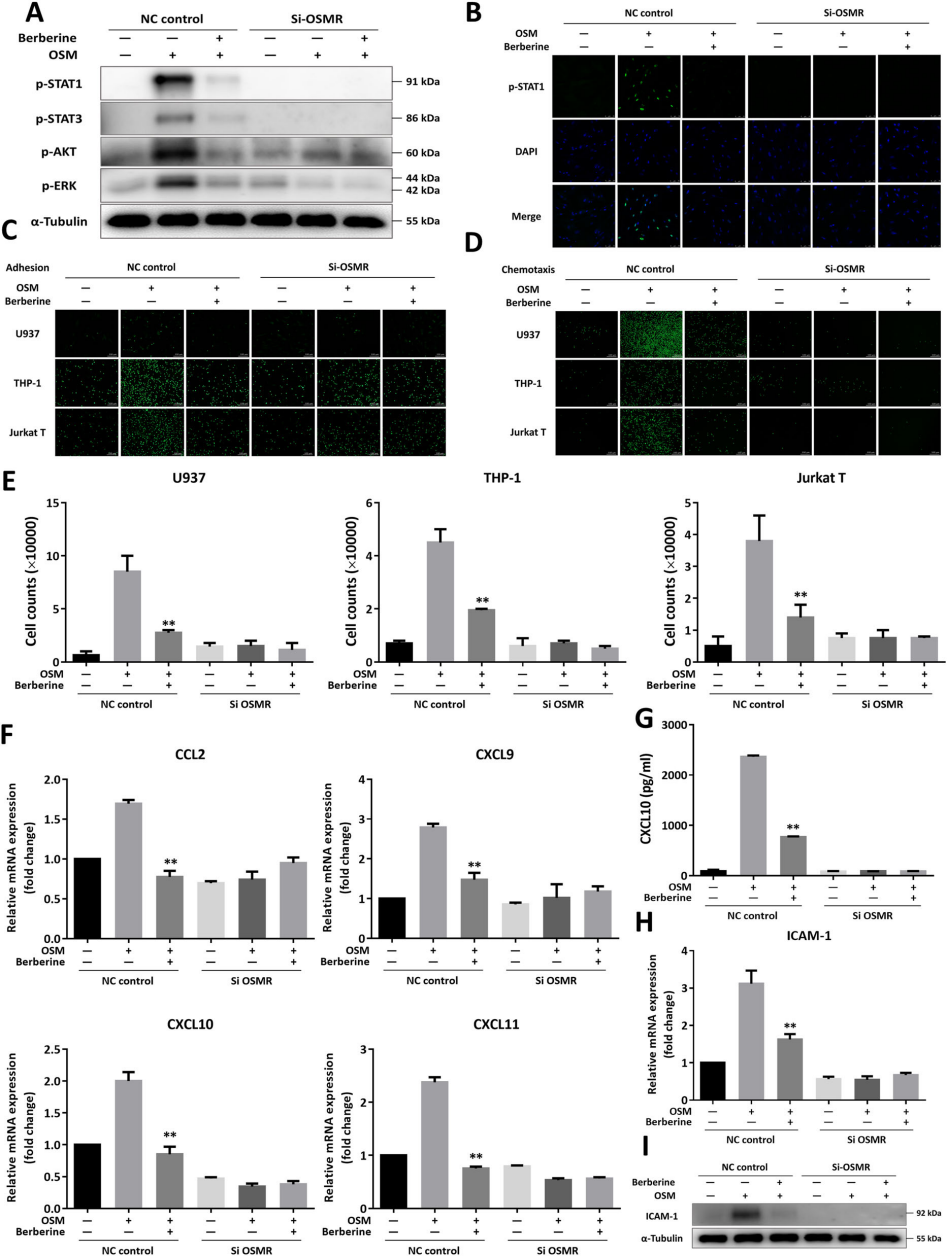
Berberine interfered with the interaction between human intestinal stromal cells and immune cells, in which OSMR functioned as an indispensible factor. **a** Human CCD-18Co cells were transfected with NC or si-OSMR for 72 h and then stimulated with OSM (10 ng/ml) for 1 h in the presence of berberine (50 μM). The cells were collected for western blot assay (**a**) and immunofluorescent staining with p-STAT1 (**b**). **c** Calcein AM-labeled U937, THP-1, and Jurkat T cells were incubated on OSM-stimulated transfected CCD-18Co cells for 30 min. Adhered immune cells were visualized under Olympus IX73 microscope. **d** Representative images of Calcein AM-labeled U937, THP-1, and Jurkat T cells migrated to the supernatants from OSM-stimulated transfected CCD-18Co cells for 2 h. **e** The counting numbers of U937, THP-1, and Jurkat T cells chemotactic to the lower chamber containing OSM-stimulated transfected CCD-18Co cells supernatants. **f** The mRNA expression of certain chemokines in OSM-stimulated transfected CCD-18Co cells. **g** Protein level of CXCL10 in OSM-stimulated transfected CCD-18Co cells. **h** The mRNA expression of ICAM-1 in OSM-stimulated transfected CCD-18Co cells. **i** Western blot assay of ICAM-1 expression in OSM-stimulated transfected CCD-18Co cells. Data were presented as mean ± SEM of three independent experiments. *p < 0.05 and **p < 0.01, compared with OSM-stimulated CCD-18Co cells, were measured by one-way ANOVA.

In depth, to uncover the critical role of OSMR in mediating the interaction between stromal cells and immune cells, we first designed three siRNA sequences targeting for decreasing OSMR expression. Due to the highest efficacy of sequence #3, we adopted it for the following investigations (Supplementary Fig. S4). Upon transfection with si-OSMR, phosphorylation of STAT1, STAT3, AKT, ERK were obviously blocked and little effects of berberine were observed in Fig. 4a, b. Next, compared to NC control, OSM was disabled to induce U937, THP-1, and Jurkat T cells adhering and migrating to transfected human CCD-18Co cells (Fig. 4c–e). Conformably, there were none effects of OSM on the expression of chemokines, CCL2, CXCL9, CXCL10, and CXCL11, and ICAM-1 in CCD-18Co cells upon si-OSMR transfection (Fig. 4f–i). In sum, it could be speculated that OSMR served as the indispensable factor mediating the interaction between intestinal stromal cells and immune cells.

### Berberine suppressed the infiltration of inflammatory cells, mucosal ILCs, and NK cells to the inflamed lamina propria in DSS-induced chronic colitis

**Fig. 5:**
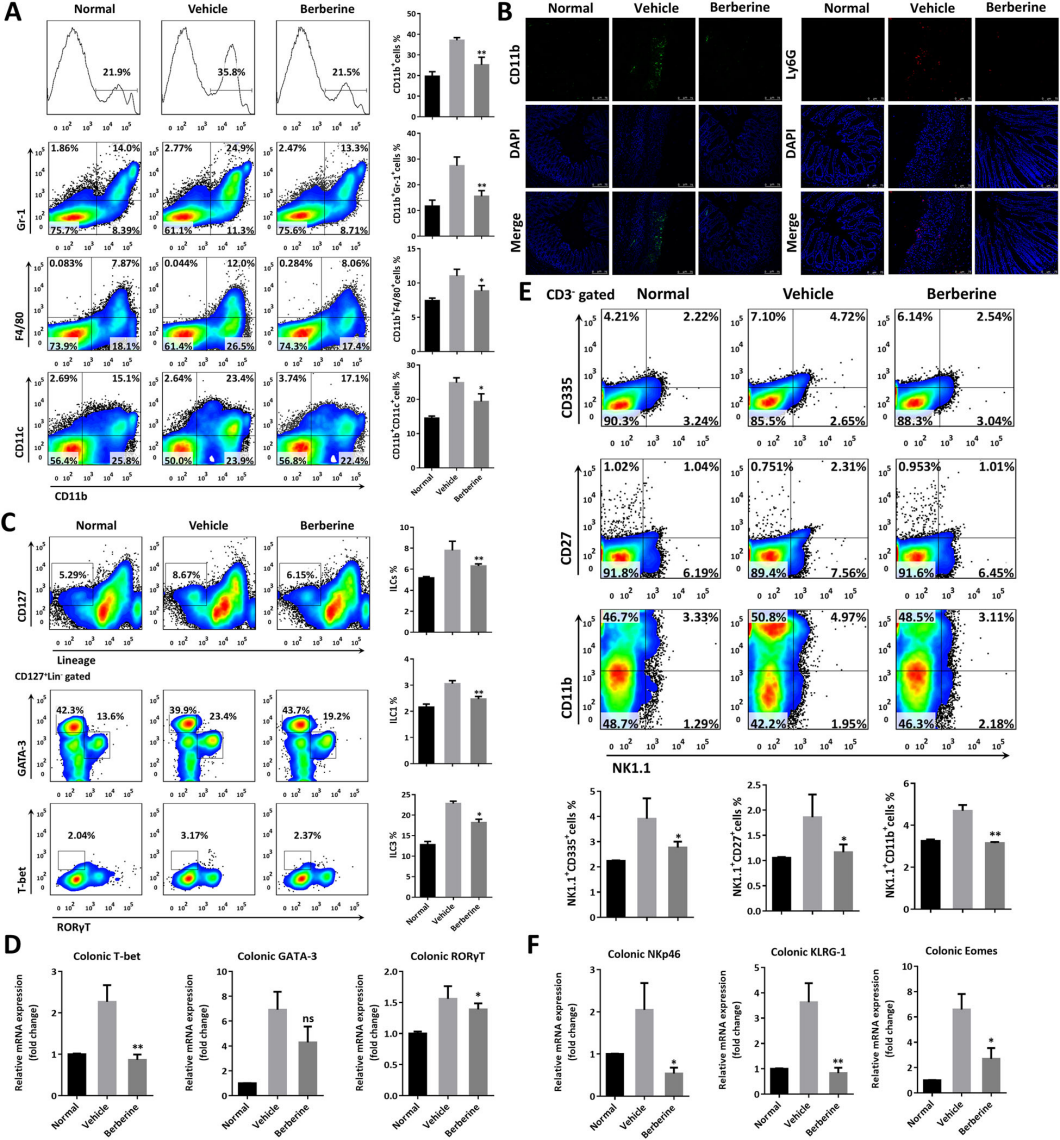
Berberine inhibited the infiltration of inflammatory cells to the inflamed colon tissue in DSS-induced chronic colitis. **a** Flow cytometry and quantitative analysis of gut lamina propria mononuclear cells (LPMCs), including monocytes (CD11b^+^), neutrophils (CD11b^+^Gr-1^+^), macrophages (CD11b^+^F4/80^+^), and dendritic cells (CD11b^+^CD11c^+^). **b** Representative images of immunofluorescence staining with CD11b and Ly6G. **c** Flow cytometry and quantitative analysis of the proportion of ILCs (CD45^+^Lineage^−^CD127^+^), including ILC1 (T-bet^+^), ILC2 (GATA3^+^), and ILC3 (RORγT^+^), in LPMCs. **d** The mRNA expression level of T-bet, GATA3, and RORγT by RT-PCR. **e** Flow cytometry and quantitative analysis of the proportion of activation of NK cells (CD335, CD27, and CD11b, gated on CD3^‒^NK1.1^+^). **f** The mRNA expression level of NKp46, KLRG-1, and Eomes by RT-PCR. Data were presented as the mean ± SEM, and *n* = 15 mice per group. *p < 0.05, and **p < 0.01, compared with the vehicle group, were measured by one-way ANOVA.

Encouraged by the above fact, local resident and pathologically new recruited leukocytes in lamina propria were analyzed by flow cytometry. As observed in Fig. 5a, the population of monocytes (CD11b^+^), neutrophils (CD11b^+^Gr-1^+^), macrophages (CD11b^+^F4/80^+^), and dendritic cells (CD11b^+^CD11c^+^) were significantly increased in DSS-treated mice, which may serve as the major source for accumulation of OSM (Fig. 5a), which drove diverse inflammatory activities in chronic UC. Immune cell infiltration was proved by immunofluorescent staining with CD11b and Ly6G, which were largely scattered over the mucosal and submucosal layers in vehicle mice, by contrast to normal controls (Fig. 5b). Energetically, berberine treatment decreased the population of neutrophils and APCs (Fig. 5a, b). Accumulating evidence implied that redundant OSM, secreted by infiltrated APCs, further enhanced leukocytes recruitment, activation, colonic retention, epithelial hyperplasia, barrier defects, and tissue remodeling^16^. Consequently, innate lymphoid cells (ILCs), identified as Lineage^−^CD127^+^, expanded in lamina propria of vehicle mice and were restored upon berberine treatment (Fig. 5c). The subsets of ILCs reflected functional categories of T helper subsets and transcriptional factors, T-bet, GATA3, and RORγT were adopted to identify ILC1, ILC2, and ILC3, respectively^31,40^. We found berberine showed ability to decrease the subpopulation of ILC1 (T-bet^+^), ILC3 (RORγT^+^), and the numbers of ILC2 (GATA3^+^) (Fig. 5c). Accordingly, berberine could inhibited the mRNA expression level of transcriptional factors, including T-bet, GATA3, and RORγT (Fig. 5d). Furthermore, compared with vehicle mice, activation of natural killer (NK) cells, indicated by the markers of CD335 (also known as NKp46), CD27, and CD11b, was also blocked following berberine treatment (Fig. 5e). Therapeutically, berberine decreased the specific transcriptional factors, including NKp46, KLRG-1, and Eomes (Fig. 5f), which were closely related to the infiltration and activation of ILCs and NK cells. In short, in line with OSM accumulation, large numbers of inflammatory cells infiltrated to the lamina propria, which were significantly decreased upon berberine treatment.

### Berberine downregulated expression of adhesive molecules, chemokines, and chemokine receptors in DSS-induced chronic colitis

**Fig. 6:**
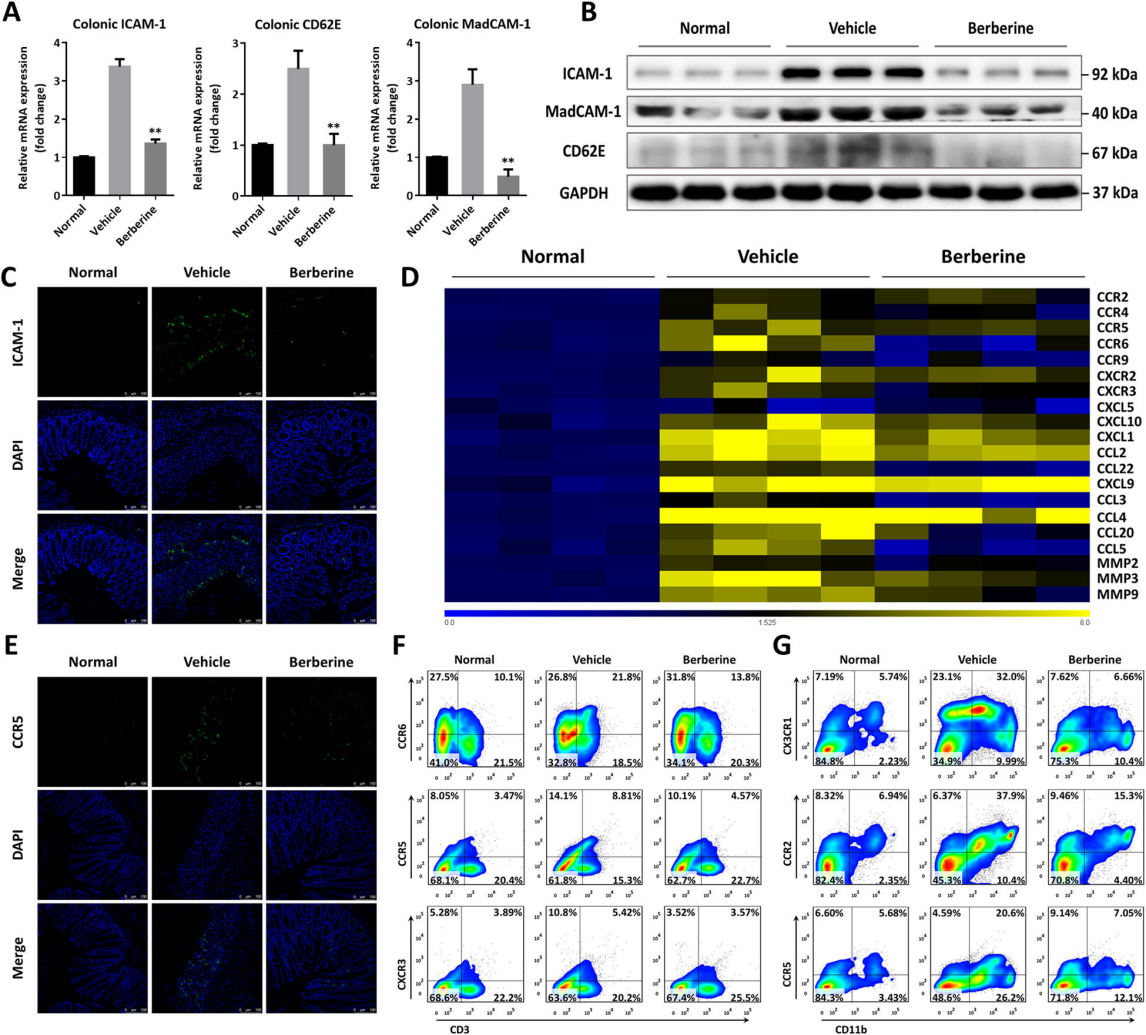
Berberine downregulated the expression levels of various adhesion molecules, chemokines, and their receptors. **a** The mRNA expression level of ICAM-1, MadCAM-1, and CD62E in colonic tissue. **b** Western blot assay of the protein expression level of ICAM-1, MadCAM-1, and CD62E in colonic tissue. **c** Representative images of immunofluorescence staining with ICAM-1. **d** Gene expression level of various chemokines and their receptors in colon tissues by RT-PCR. **e** Representative images of immunofluorescence staining with CCR5. **f** Flow cytometry analysis of CCR5, CXCR3, and CCR6, gated on CD3^+^ cells. **g** Flow cytometry analysis of CCR5, CCR2, and CX3CR1, gated on CD11b^+^ cells. Data were presented as the mean ± SEM, and *n* = 15 mice per group. *p < 0.05 and **p < 0.01, compared with the vehicle group, were measured by one-way ANOVA.

To further explore the underlying mechanisms, along with OSM induction, gene and protein expression assays were performed to measure the level of adhesive molecules, chemokines, and chemokine receptors. Expectedly, compared with normal controls, we observed increased mRNA and protein expression of several adhesive molecules, including ICAM-1, MadCAM-1, and CD62E (E-selectin), which were suppressed when colitic mice treated with berberine (Fig. 6a, b). Further in situ immunofluorescent staining in Fig. 6c suggested ICAM-1 was largely expressed around the region of epithelium and basement layer, which contributed to recruiting inflammatory cells from circulatory system and/or lymphoid organs. In addition, berberine downregulated the mRNA expression level of multiple matrix metalloproteinases, chemokines, and their corresponding receptors, such as MMP9, CCL2, CCL3, CXCL10, CCR5, CCR6, and CXCR3, which were affirmed by immunofluorescent staining (Fig. 6d, e). Consistently, increased expression of CXCR3, CCR5, and CCR6 on CD3^+^ cells, as well as CCR5, CCR2, and CX3CR1 on CD11b^+^ cells were exhibited in colonic lamina propria of DSS-treated mice, which were attenuated following berberine treatment (Fig. 6f, g).

## Discussion

UC manifested as an idiopathic, relapsing and etiologically complicated inflammatory disorder, involving the interaction between environment factors, genetic emotivity, perturbation of immune homeostasis, and dysfunction of intestinal mucosal barrier^5,6,18^. Over the past decades, oral administration of berberine has been demonstrated to exert protective capacity on DSS-induced experimental acute UC, which dominantly attributed to the critical role of berberine in modulating the inflammatory responses from immune cell and epithelial cells, maintaining the intestinal barrier function, and regulating gut microbiota^24,41,42^. Besides, berberine could also attenuate colonic macromorphological and histopathological inflammation through suppressing the expansion and overaction of Th17 cells in DSS-induced murine chronic UC^43,44^. Moreover, the phase 4 clinical investigations from Xijing Hospital of Digestive Diseases have been projected and designed to examine the therapeutic effects of berberine on the annual recurrence rate of UC in remission (NCT02962245, ClinicalTrials.gov).

However, upon oral administration, berberine undergoes multiple metabolic pathways, including demethylation, reduction, and hydroxylation, which lead to an extreme low systemic exposure^45,46^. Due to CYP450 metabolism, interaction with gut microbiota, along with production of butyrate, berberine could be converted into its absorbable or active forms, such as berberrubine, dihydroberberine, and the demethyleneberberine^45,47^. Nevertheless, dihydroberberine was elucidated to be unstable in solution and reverted into berberine largely via non-enzymatic oxidation in intestinal tissues^45^. Wang et al. also demonstrated that berberine was relatively stable in the gut lumen and the remained berberine could interact with the gut microbiota, accounting for the effects of berberine in UC remission^42,45^. Recently, gut microbiota-modulated pharmacokinetics in beagle dogs suggested that berberine and dihydroberberine could be detected with high excreted amounts in the feces samples, but a low content in plasma^48^. From another point of view, the parent nucleus of quaternary ammonium was indeed reserved during multiple metabolic processes, which might collectively provide a similar molecular mechanism in ameliorating UC.

Although the underlying molecular mechanism of berberine in chronic intestinal mucosal inflammation remains ill-defined, the current research described was intended to further understand the role of berberine in DSS-induced murine chronic UC. It has been demonstrated that overexpression of OSM was closely associated with gut inflammation and disease severity of IBD. Consistently, OSM^−^^/−^ mice displayed reduced colonic pathology and OSM-associated inflammatory modules in the experimental colitis attributing to *H. hepaticus* and α-IL-10R exposure (*Hh*+α-IL-10R colitis). Moreover, OSMR fusion protein (OR-Fc), neutralizing the function of OSM, also distinctly prevented disease severity and mucosal inflammatory responses in *Hh* and α-IL-10R delivered wild-type mice^15^. A chronic relapsing colitis model induced by DSS repeated exposure could clinically mimic key histopathological features of UC in human^26^. Increasingly, we first identified an increased expression of OSM and OMSR in the inflamed colonic tissue of DSS-induced murine chronic UC (Fig. 3), which pathologically accounted for the following inflammatory damage, intestinal fibrosis, and immune cells recruitment.

OSM, referring to the cytokine family of IL-6 and secreted by neutrophils, APCs and T cells, induces inflammatory genes expression and contributes to mucosal inflammation and tissue damage, which has a vital role in numerous chronic diseases^13^. In the present research, we found berberine could dose-dependently inhibited OSM production from T cells, BMDMs, and BMDCs, induced by TCR crosslinking and LPS, respectively (Supplementary Fig. S1), whereas, there is little effect of berberine on IL-6 from the in vitro study, which encouraged us to focus on the molecular mechanism of berberine by interfering with OSM-mediated signaling in treating UC. In accordance with previous report, berberine treatment effectively alleviated the experimental symptoms and mucosal inflammation of DSS-induced chronic UC, as evidenced by disease severity scores, histological examination, and evaluation of inflammatory responses in the colonic tissue (Fig. 1). In addition, breakdown of the intestinal barrier underpins the development of UC^37,49,50^ and berberine could protected the mucosal barrier integrity and function from damage (Fig. 2). Reduction of microvillar structures, enlarged tight junctions and dysfunction of organelle vacuoles were observed in vehicle mice from TCM morphology (Fig. 2b). Consistently, abnormal expression of Tj proteins and MUC2 manifested in the colitic mice and berberine showed capacity to rectify these pathological changes (Fig. 2).

Intestinal fibrosis serves as the key pathological determinant in the progression of chronic UC, which referred to overactivation of intestinal stromal cells, persistent depositions of collagen, and thickening of intestinal wall^10,51^. OSM augment resulted in the overexpression of FAP and PDPN in OSMR^+^ stromal cells, which contributed to intestinal fibrosis of IBD patients^15^. In the mucosal and submucosal layers, deposits of collagen and increased expression of α-SMA were proven in our research, which was restored upon berberine treatment (Fig. 3a, b). Markedly, FAP and PDPN were highly increased in the inflamed tissue of DSS-induced chronic colitis, which might be a hint for the pathogenesis of intestinal fibrosis and the underlying mechanism of berberine (Fig. 3c). As illustrated in Fig. 3, in line with the experimental manifestations, berberine treatment obviously attenuated deposition of collagens and excessive proliferation of stromal cells, which coincided with the fact that berberine decreased the expression of OSM and OSMR in inflamed colonic homogenates and explants, serum, stool extracts (Fig. 3e–g).

In addition to the role of stromal cells in fibrosis, overexpression of adhesive factors and chemokines by stromal cells have also been verified following OSM accumulation^52,53^. Accordingly, results from mRNA expression suggested that various adhesive molecules and chemokines were upregulated upon DSS exposure, such as ICAM-1, CCL2, and CXCL10 (Fig. 6). As a consequence, a certain number of neutrophils, APCs, ILCs, and activated NK cells were infiltrated to the inflamed tissue, which inevitably resulted in further enrichment of inflammatory cytokines pools, barrier disturbance, and intestinal fibrosis (Fig. 5). Berberine treatment demonstrated modulatory effects on inflammatory infiltrations accompanied by suppressing the expression of adhesive molecules and chemokines.

**Fig. 7:**
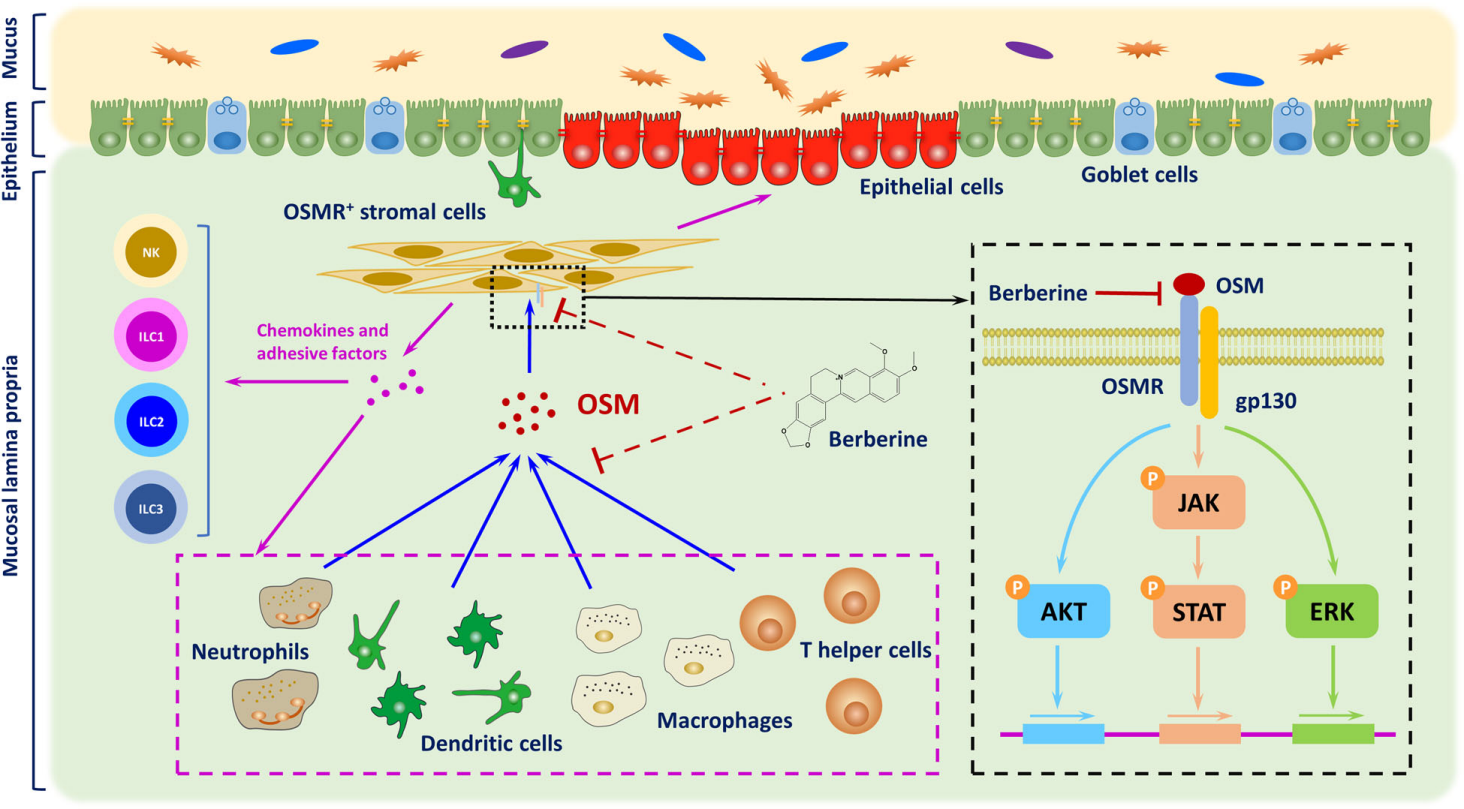
Diagram of OSM-driven intestinal mucosal inflammation interfered by berberine in treating chronic UC. In the development of chronic UC, dysfunction of intestinal barrier and rouse of gut resident immune cells contributed to initiating pathogenic colitis. Further activation of resident and newly infiltrated immune cells, including neutrophils, dendritic cells, macrophages, Th cell, led to accumulation of OSM, which subsequently induced diverse inflammatory responses in OSMR-expressing stromal cells. Berberine could suppress multiple sources of OSM and activation of stromal cells, accounting for decreased expression of chemokines, adhesive molecules, and persistent damage to epithelium homeostasis. In depth, berberine impeded the phosphorylation of JAK-STAT, ERK, and AKT signaling through interfering with the binding mode of OSM and OSMR.

Collectively, berberine could not only interfere with the production of OSM from activated neutrophils, dendritic cells, macrophages, and T cells, but also impeded OSM-driven activation of stromal cells and recruitment of immune cells (Fig. 7). To deeply understand the molecular mechanism of berberine in mucosal inflammation, human intestinal stromal cells, CCD-18Co cells, were included in our study to uncover the role of berberine in modulating the interaction between stromal cells and immune cells. We found that OSM addition significantly led to recruitment of monocytes and T cells via increasing expression of chemokines and ICAM-1 (Fig. 4), which were in accordance with in vivo results (Fig. 6). Berberine could dose-dependently weaken the interaction between stromal cells and immune cells, which ascribed suppression of phosphorylation of STAT1, STAT3, AKT, and ERK (Fig. 3 and Supplementary Fig. S3). In depth, recruitment of immune cells was extremely blocked in OSM-stimulated CCD-18Co cells upon knockdown of OSMR (Fig. 4).

In conclusion, the current research demonstrated that oral administration of berberine exerted therapeutic effects in experimental chronic UC, manifested with attenuating gut inflammation, protecting intestinal barrier function, restoring tissue remodeling and fibrosis, and decreasing inflammatory infiltrations. Accordingly, the effects were closely associated with OSM-mediated JAK-STAT, MAPK, and AKT signaling through OSMR (Fig. 7). Our study provided an infusive molecular mechanism of berberine and suggested OSM and OSMR intervention might function as the potential therapeutic target in chronic UC.

## Supplementary information

Supplementary information

Supplementary Figure 1

Supplementary Figure 2

Supplementary Figure 3

Supplementary Figure 4
