# Supplementary material for: Intervention of oncostatin M-driven mucosal inflammation by berberine exerts therapeutic property in chronic ulcerative colitis
Source: Cell Death Dis. 2020 Apr 24;11(4):271. doi: 10.1038/s41419-020-2470-8 (PMC7181765; doi:10.1038/s41419-020-2470-8)
Supplement: Supplementary file 1 — Supplementary information [file 41419_2020_2470_MOESM1_ESM.doc]

**Supplementary Information**

**Title: Intervention of oncostatin M-driven mucosal inflammation by berberine exerts therapeutic property in chronic ulcerative colitis**

Heng Li^1, 2^, Chunlan Feng^1^, Chen Fan^1^, Yang Yang^3^, Xiaoqian Yang^1^, Huimin Lu^1, 2^, Qiukai Lu^1, 2^, Fenghua Zhu^1^, Caigui Xiang^1, 2^, Zongwang Zhang^1^, Peilan He^1^, Jianping Zuo^1, 2, 3^ *, Wei Tang^1, 2^, *

^1^ Laboratory of Anti-inflammation and Immunopharmacology, Shanghai Institute of Materia Medica, Chinese Academy of Sciences, Shanghai 201203, China

^2^ School of Pharmacy, University of Chinese Academy of Sciences, Beijing 100049, China

^3^ Laboratory of Immunology and Virology, Shanghai University of Traditional Chinese Medicine, Shanghai 201203, China

* **Address for correspondence:**

E-mail: tangwei@simm.ac.cn (Wei Tang), and jpzuo@simm.ac.cn (Jianping Zuo). Tel: +86-21-50806820 (Wei Tang), and +86-21-50806701 (Jianping Zuo).

**Author Contributions**

H Li, W Tang and JP Zuo contributed to the conception, design of the study, analysis and interpretation of data. H Li, CL Feng, C Fan, QK Lu, HM Lu, Y Yang, XQ Yang, CG Xiang, FH Zhu, ZW Zhang, and PL He performed the experiments and acquired the data. W Tang and JP Zuo revised the manuscript critically for important intellectual content. All authors approved the final version of the manuscript.

**Supplementary Fig. S1. Berberine suppressed OSM secretion from *ex vivo* and *in vitro* stimulation of immune cells.** (A) Splenocytes from three groups were prepared and stimulated with anti-CD3 or LPS for 48 h. The supernatants were assayed for OSM by ELISA. (B) Purified CD4^+^ T cells from spleen were stimulated with anti-CD3 and anti-CD28 for 48 h and then assayed for OSM by ELISA. (C) MLNs were prepared and stimulated with anti-CD3 or LPS for OSM detection. (D) Purified CD4^+^ T cells from MLNs were stimulated with anti-CD3 and anti-CD28 for OSM detection. (E) Purified CD4^+^ T cells from naïve mice were incubated with berberine (10, 5, and 2.5 μM) for cell viability assay and were stimulated with anti-CD3 and anti-CD28 for OSM detection. (F) BMDMs were incubated with berberine (10, 5, and 2.5 μM) for cell viability assay and stimulated with LPS for 24 h for OSM detection. (G) BMDCs were incubated with berberine (10, 5, and 2.5 μM) for cell viability assay and stimulated with LPS for 24 h for OSM detection. (A)-(D) Data were presented as the mean±SEM, and n=15 mice per group. *p<0.05, and **p<0.01, compared with the vehicle group, were measured by one-way ANOVA. (E)-(G) Data were presented as mean±SEM of three independent experiments. *p<0.05, and **p<0.01, compared with activated immune cells group, were measured by one-way ANOVA.

**Supplementary Fig. S2. Berberine suppressed the phosphorylation of STAT1 and STAT3 in the colonic lamina propria of DSS-induced chronic colitis.** (A) Representative images of flow cytometry of phosphorylation of STAT1 and STAT3 on CD3^+^ cells, prepared from colonic lamina propria. (B) Representative images of flow cytometry of phosphorylation of STAT1 and STAT3 on CD11b^+^ cells, prepared from colonic lamina propria.

**Supplementary Fig. S3. Berberine dose-dependently inhibited immune cells infiltration to OSM-driven human intestinal stromal cells.** (A) Primary human intestinal stromal cells, CCD-18Co cells, were incubated with berberine (50, 25, and 12.5 μM) for cell viability assay. (B) Calcein AM-labeled U937, THP-1, and Jurkat T cells were incubated on CCD-18Co cells and the adhered cells were visualized under Olympus IX73 microscope. (C) The counting numbers of U937, THP-1, and Jurkat T cells chemotactic to the lower chamber containing OSM-stimulated CCD-18Co cells supernatants. (D) Representative images of Calcein AM-labeled U937, THP-1, and Jurkat T cells migrated to the supernatants from OSM-stimulated CCD-18Co cells. (E) The mRNA expression of ICAM-1 and chemokines in OSM-stimulated CCD-18Co cells. (F) Western blot assay of phosphorylation of STAT1, STAT3, AKT, and ERK in OSM-stimulated CCD-18Co cells. Data were presented as mean±SEM of three independent experiments. *p<0.05, and **p<0.01, compared with OSM-stimulated CCD-18Co cells, were measured by one-way ANOVA.

**Supplementary Fig. S4. The percentage of knockdown for OSMR in human CCD-18Co cells.** Human CCD-18Co cells were transfected with three si-OSMR sequences for 72 h and then collected for western blot assay (A), RT-PCR visualized by 1% agarose gel (B), and quantitative real-time PCR (C). Data were presented as mean±SEM of three independent experiments. *p<0.05, and **p<0.01, compared with NC-transfected CCD-18Co cells, were measured by Student’s t test.

**Supplementary Tab. S1 Sequences of primers for quantitative RT-PCR**

| **Gene** | **Sequence 5′-3′** | | | |
| --- | --- | --- | --- | --- |
| **Mouse** |  |  |  |  |
| **β-actin** | F | GGCTGTATTCCCCTCCATCG | R | CCAGTTGGTAACAATGCCATGT |
| **MUC2** | F | AGGGCTCGGAACTCCAGAAA | R | CCAGGGAATCGGTAGACATCG |
| **ZO-1** | F | GCCGCTAAGAGCACAGCAA | R | TCCCCACTCTGAAAATGAGGA |
| **E-cadherin** | F | CAGGTCTCCTCATGGCTTTGC | R | CTTCCGAAAAGAAGGCTGTCC |
| **Occludin** | F | TTGAAAGTCCACCTCCTTACAGA | R | CCGGATAAAAAGAGTACGCTGG |
| **OSM** | F | ATGCAGACACGGCTTCTAAGA | R | TTGGAGCAGCCACGATTGG |
| **OSMR** | F | CATCCCGAAGCGAAGTCTTGG | R | GGCTGGGACAGTCCATTCTAAA |
| **FAP** | F | GTCACCTGATCGGCAATTTGT | R | CCCCATTCTGAAGGTCGTAGAT |
| **PDPN** | F | ACCGTGCCAGTGTTGTTCTG | R | AGCACCTGTGGTTGTTATTTTGT |
| **T-bet** | F | AGCAAGGACGGCGAATGTT | R | GGGTGGACATATAAGCGGTTC |
| **GATA3** | F | CTCGGCCATTCGTACATGGAA | R | GGATACCTCTGCACCGTAGC |
| **RORγT** | F | GACCCACACCTCACAAATTGA | R | AGTAGGCCACATTACACTGCT |
| **NKp46** | F | ATGCTGCCAACACTCACTG | R | GATGTTCACCGAGTTTCCATTTG |
| **KLRG-1** | F | TTTGGGGCTTTTGACTGTGAT | R | TGTAAGGAGATGTGAGCCTTTGT |
| **Eomes** | F | GCGCATGTTTCCTTTCTTGAG | R | GGTCGGCCAGAACCACTTC |
| **ICAM-1** | F | GTGATGCTCAGGTATCCATCCA | R | CACAGTTCTCAAAGCACAGCG |
| **MadCAM-1** | F | CCTGGCCCTAGTACCCTACC | R | CCGTACAGAGAGGATACTGCTG |
| **CD62E** | F | ATGCCTCGCGCTTTCTCTC | R | GTAGTCCCGCTGACAGTATGC |
| **MMP2** | F | CAAGTTCCCCGGCGATGTC | R | TTCTGGTCAAGGTCACCTGTC |
| **MMP3** | F | ACATGGAGACTTTGTCCCTTTTG | R | TTGGCTGAGTGGTAGAGTCCC |
| **MMP9** | F | CTGGACAGCCAGACACTAAAG | R | CTCGCGGCAAGTCTTCAGAG |
| **CCR2** | F | ATCCACGGCATACTATCAACATC | R | CAAGGCTCACCATCATCGTAG |
| **CCR4** | F | GGAAGGTATCAAGGCATTTGGG | R | GTACACGTCCGTCATGGACTT |
| **CCR5** | F | TTTTCAAGGGTCAGTTCCGAC | R | GGAAGACCATCATGTTACCCAC |
| **CCR6** | F | CCTGGGCAACATTATGGTGGT | R | CAGAACGGTAGGGTGAGGACA |
| **CCR9** | F | CTTCAGCTATGACTCCACTGC | R | CAAGGTGCCCACAATGAACA |
| **CXCR2** | F | ATGCCCTCTATTCTGCCAGAT | R | GTGCTCCGGTTGTATAAGATGAC |
| **CXCR3** | F | TACCTTGAGGTTAGTGAACGTCA | R | CGCTCTCGTTTTCCCCATAATC |
| **IP-10** | F | CCAAGTGCTGCCGTCATTTTC | R | GGCTCGCAGGGATGATTTCAA |
| **KC** | F | CTGGGATTCACCTCAAGAACATC | R | CAGGGTCAAGGCAAGCCTC |
| **MCP-1** | F | TTAAAAACCTGGATCGGAACCAA | R | GCATTAGCTTCAGATTTACGGGT |
| **MDC** | F | AGGTCCCTATGGTGCCAATGT | R | CGGCAGGATTTTGAGGTCCA |
| **MIG** | F | TCCTTTTGGGCATCATCTTCC | R | TTTGTAGTGGATCGTGCCTCG |
| **MIP-1α** | F | TTCTCTGTACCATGACACTCTGC | R | CGTGGAATCTTCCGGCTGTAG |
| **MIP-1β** | F | TTCCTGCTGTTTCTCTTACACCT | R | CTGTCTGCCTCTTTTGGTCAG |
| **MIP-3α** | F | ACTGTTGCCTCTCGTACATACA | R | GAGGAGGTTCACAGCCCTTTT |
| **RANTES** | F | GCTGCTTTGCCTACCTCTCC | R | TCGAGTGACAAACACGACTGC |
| **ENA-78** | F | TCCAGCTCGCCATTCATGC | R | TTGCGGCTATGACTGAGGAAG |
| **Human** |  |  |  |  |
| **GAPDH** | F | GGAGCGAGATCCCTCCAAAAT | R | GGCTGTTGTCATACTTCTCATGG |
| **CCL2** | F | CAGCCAGATGCAATCAATGCC | R | TGGAATCCTGAACCCACTTCT |
| **CCL3** | F | AGTTCTCTGCATCACTTGCTG | R | CGGCTTCGCTTGGTTAGGAA |
| **CCL4** | F | CTGTGCTGATCCCAGTGAATC | R | TCAGTTCAGTTCCAGGTCATACA |
| **CCL17** | F | ATGGCCCCACTGAAGATGCT | R | TGAACACCAACGGTGGAGGT |
| **CCL20** | F | TGCTGTACCAAGAGTTTGCTC | R | CGCACACAGACAACTTTTTCTTT |
| **CXCL9** | F | CCAGTAGTGAGAAAGGGTCGC | R | AGGGCTTGGGGCAAATTGTT |
| **CXCL10** | F | GTGGCATTCAAGGAGTACCTC | R | TGATGGCCTTCGATTCTGGATT |
| **CXCL11** | F | GACGCTGTCTTTGCATAGGC | R | GGATTTAGGCATCGTTGTCCTTT |
| **ICAM-1** | F | ATGCCCAGACATCTGTGTCC | R | GGGGTCTCTATGCCCAACAA |

**Supplementary Tab. S2 Antibodies for western blot assay**

| **Antibody** | **Vendor** | **Catalog No.** |
| --- | --- | --- |
| HRP anti-GAPDH | Proteintech | HRP-60004 |
| anti-Occludin | Thermo Fisher Scientific | 71-1500 |
| anti-E-cadherin | Cell Signaling Technology | 3195 |
| anti-ZO-1 | Proteintech | 21773-1-AP |
| anti-MUC2 | Novus | NB120-11197 |
| anti-Claudin-1 | Abcam | ab15098 |
| anti-Claudin-2 | Abcam | ab15104 |
| anti-α-SMA | Cell Signaling Technology | 19245 |
| anti-NLRP3 | Cell Signaling Technology | 15101 |
| anti-ASC | Proteintech | 10500-1-AP |
| anti-Caspase-1 | Abcam | ab179515 |
| anti-ICAM-1 | Abcam | ab179707 |
| anti-CD62E | Abcam | ab18981 |
| anti-MadCAM-1 | Abcam | ab198277 |
| anti-OSM | Raybiotech | 144-06163-200 |
| anti-OSMR | Thermo Fisher Scientific | MA5-23921 |
| anti-p-STAT1 | Cell Signaling Technology | 7649 |
| anti-p-STAT3 | Cell Signaling Technology | 9145 |
| anti-p-STAT4 | Cell Signaling Technology | 5267 |
| anti-p-STAT5 | Cell Signaling Technology | 9351 |
| anti-p-STAT6 | Cell Signaling Technology | 56554 |
| anti-p-JAK1 | Cell Signaling Technology | 74129 |
| anti-p-JAK2 | Cell Signaling Technology | 3776 |
| anti-p-ERK | Cell Signaling Technology | 4377 |
| anti-p-Akt | Cell Signaling Technology | 4056 |
