# Supplementary figures and images for: Intervention of oncostatin M-driven mucosal inflammation by berberine exerts therapeutic property in chronic ulcerative colitis
Source: Cell Death Dis. 2020 Apr 24;11(4):271. doi: 10.1038/s41419-020-2470-8 (PMC7181765; doi:10.1038/s41419-020-2470-8)

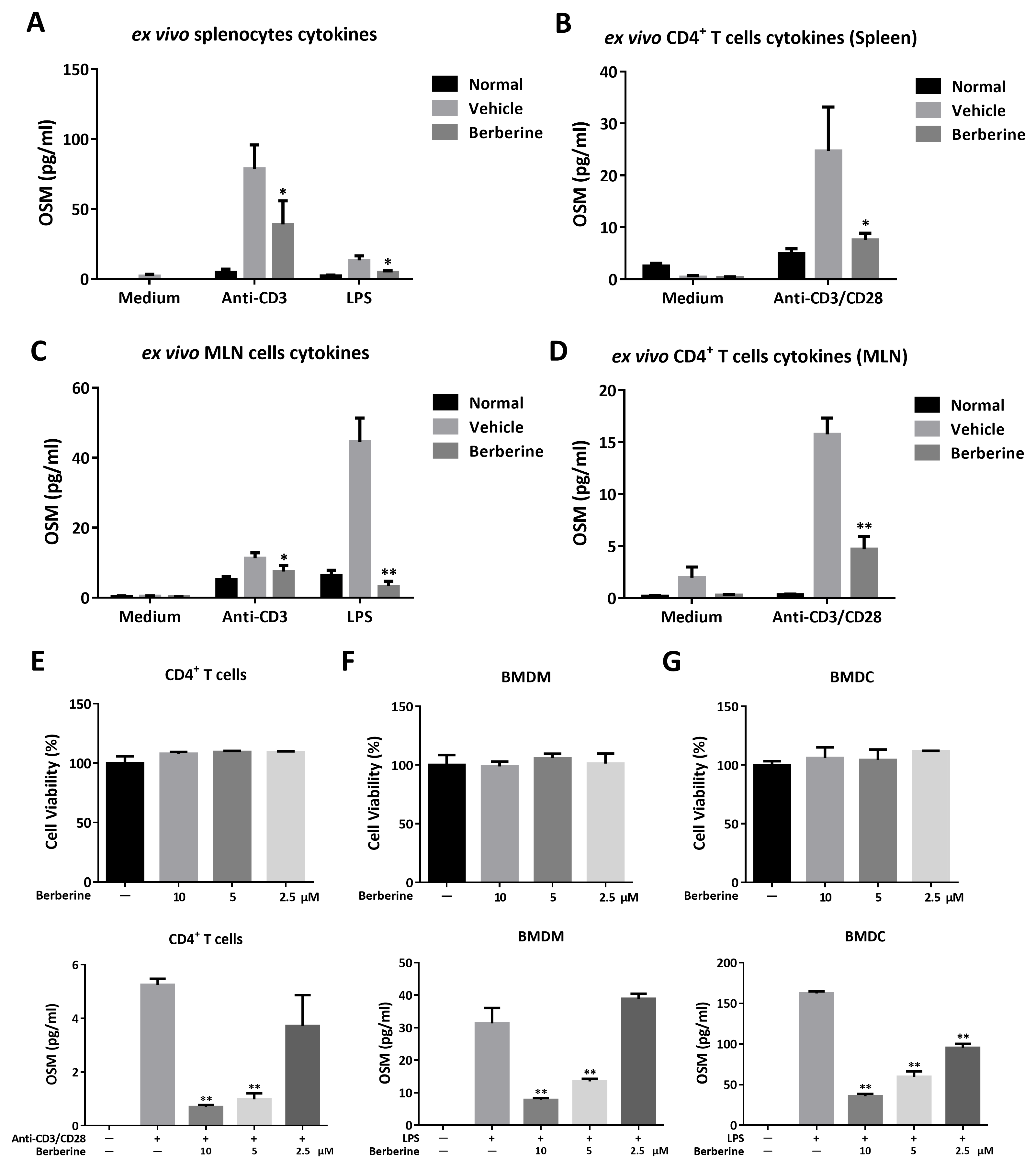

Supplement: Supplementary file 2 — Supplementary Figure 1 [file 41419_2020_2470_MOESM2_ESM.tif]

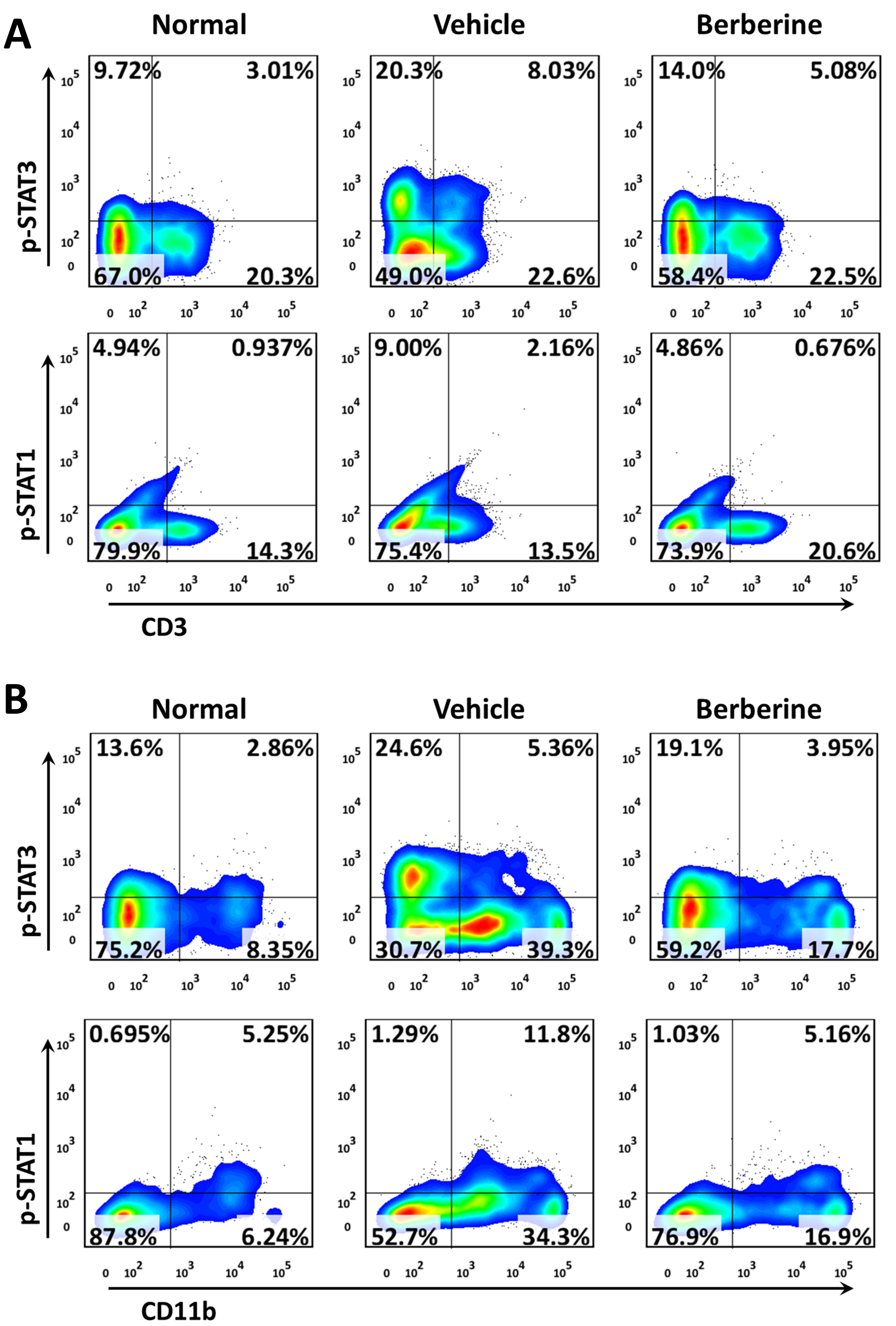

Supplement: Supplementary file 3 — Supplementary Figure 2 [file 41419_2020_2470_MOESM3_ESM.tif]

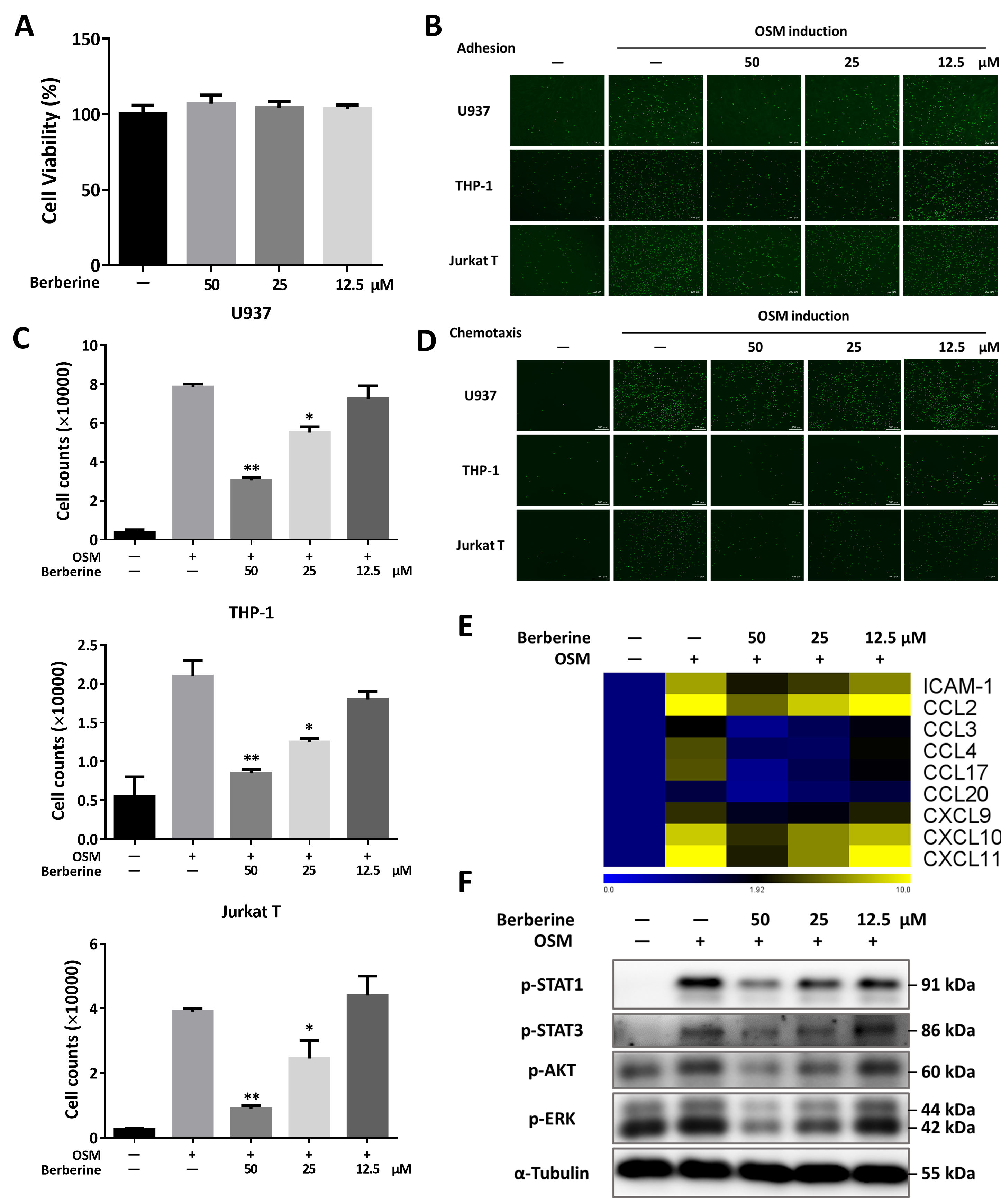

Supplement: Supplementary file 4 — Supplementary Figure 3 [file 41419_2020_2470_MOESM4_ESM.tif]

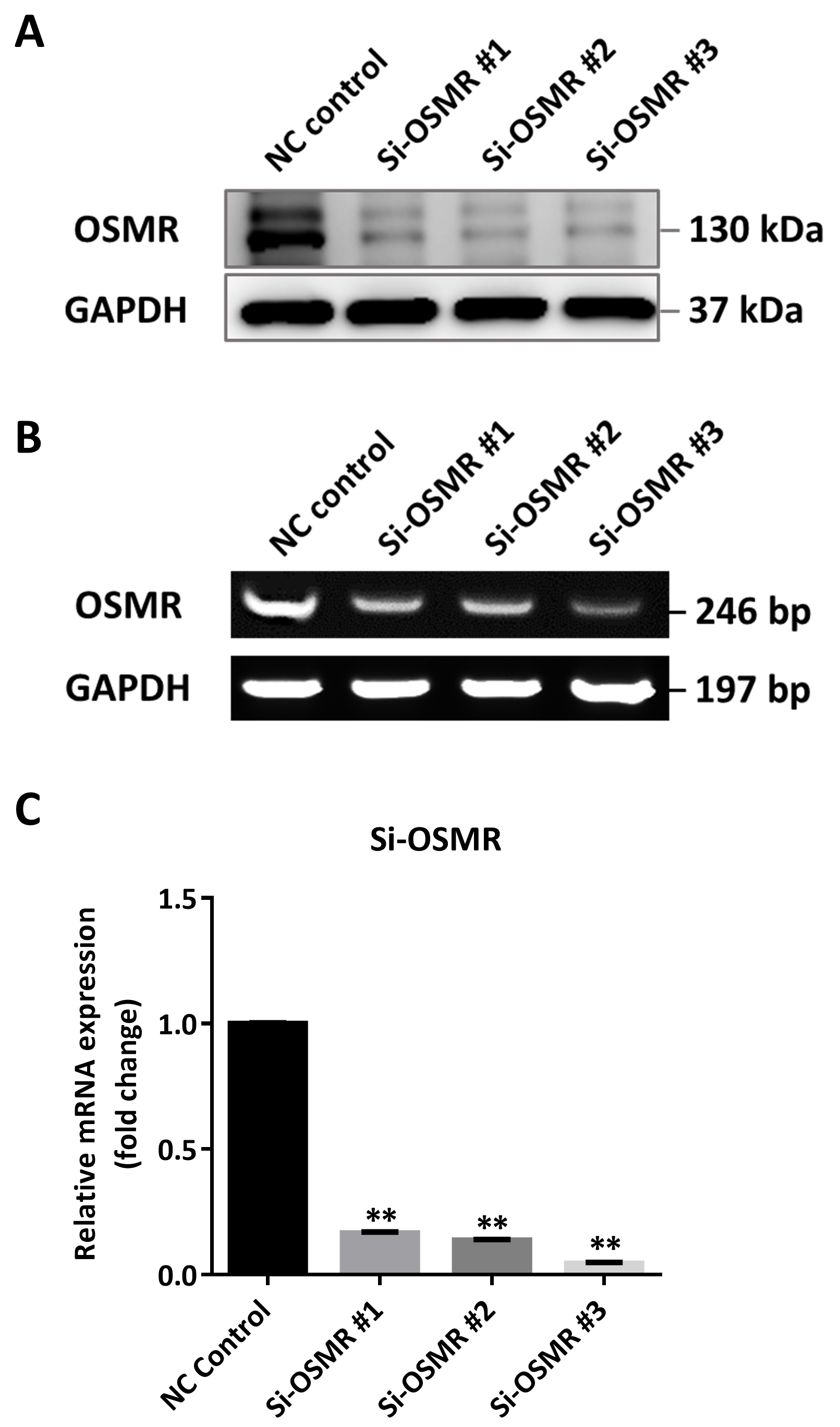

Supplement: Supplementary file 5 — Supplementary Figure 4 [file 41419_2020_2470_MOESM5_ESM.tif]
